# Optimization of pulsed electric field pretreatment followed by cellulase hydrolysis for enhanced protein recovery and antioxidant activity from brown seaweed (*Undaria pinnatifida*)

**DOI:** 10.1186/s40643-026-01140-2

**Published:** 2026-09-22

**Authors:** Antony Rinthiya Mariadavid, Maya Malikhaturohmah, Hye-Ryeon An, Sana Mansoor, Giovanni Luzi, Jae Hak Sohn, Khawaja Muhammad Imran Bashir, Jae-Suk Choi

**Affiliations:** 1 Department of Seafood Science and Technology, The Institute of Marine Industry, Gyeongsang National University, Tongyeong, 53064 Republic of Korea; 2 LSTME-Busan, 31, Gwahaksandan 1-ro, 60 beon-gil, Gangseo-gu, Busan, 46742 Republic of Korea; 3 Department of Food Science and Culinary Arts, College of Health and Welfare, Silla University, Busan, 46958 Republic of Korea

**Keywords:** Non-thermal processing, Electroporation, Cell membrane disruption, Enzymatic hydrolysis, Marine biorefinery, Antioxidant activity, Energy-efficient bioprocessing

## Abstract

*Undaria pinnatifida* is a promising brown seaweed due to its relatively high protein content and functional bioactive compounds. However, efficient recovery of these components is limited by its rigid, polysaccharide-rich cell wall structure. In this study, pulsed electric field (PEF) treatment followed by cellulase hydrolysis was investigated as a sequential approach to enhance protein extraction and antioxidant functionality from *U. pinnatifida*. PEF conditions were optimized by varying electric field strength, pulse width, pulse repetition frequency, treatment duration, seaweed loading, and pretreatment conditions to maximize protein release. The optimized conditions were 3 kV cm^−1^ electric field strength, 10 µs pulse width, 100 Hz pulse repetition frequency, and 60 s treatment duration, using 100 g of seaweed pretreated by mechanical cutting followed by 2 days of soaking in 3.5% saline solution. Under these conditions, protein concentration reached 493 ± 2.06 µg mL^−1^. The optimized PEF treatment required 0.18 kJ of electrical energy and achieved an energy efficiency of 684.72 ± 2.78 mg protein kJ^−1^. Subsequent cellulase hydrolysis further enhanced protein extraction, with the PEF + 1% cellulase treatment yielding 1307 ± 38.62 µg mL^−1^, compared with 870 ± 42.38 µg mL^−1^ for 1% cellulase alone, 512 ± 15.94 µg mL^−1^ for PEF alone, and 275 ± 10.46 µg mL^−1^ for the untreated control. Thus, the combined treatment produced approximately 50%, 155%, and 375% higher protein concentrations than 1% cellulase alone, PEF alone, and the untreated control, respectively. Antioxidant activity assessed separately using DPPH, ABTS, and FRAP assays also showed the highest responses for the combined treatment, with 52.08 ± 2.68% DPPH radical-scavenging activity, 51.00 ± 1.08% ABTS radical-scavenging activity, and 679 ± 1.08 µM Fe^2+^ equivalents in the FRAP assay. Overall, the optimized sequential PEF–cellulase treatment substantially improved protein extraction and produced the highest antioxidant responses among the treatments investigated, demonstrating its potential for enhancing the recovery of protein and antioxidant-active components from *U. pinnatifida* under laboratory-scale conditions.

**Graphical abstract:**

**Figure:**
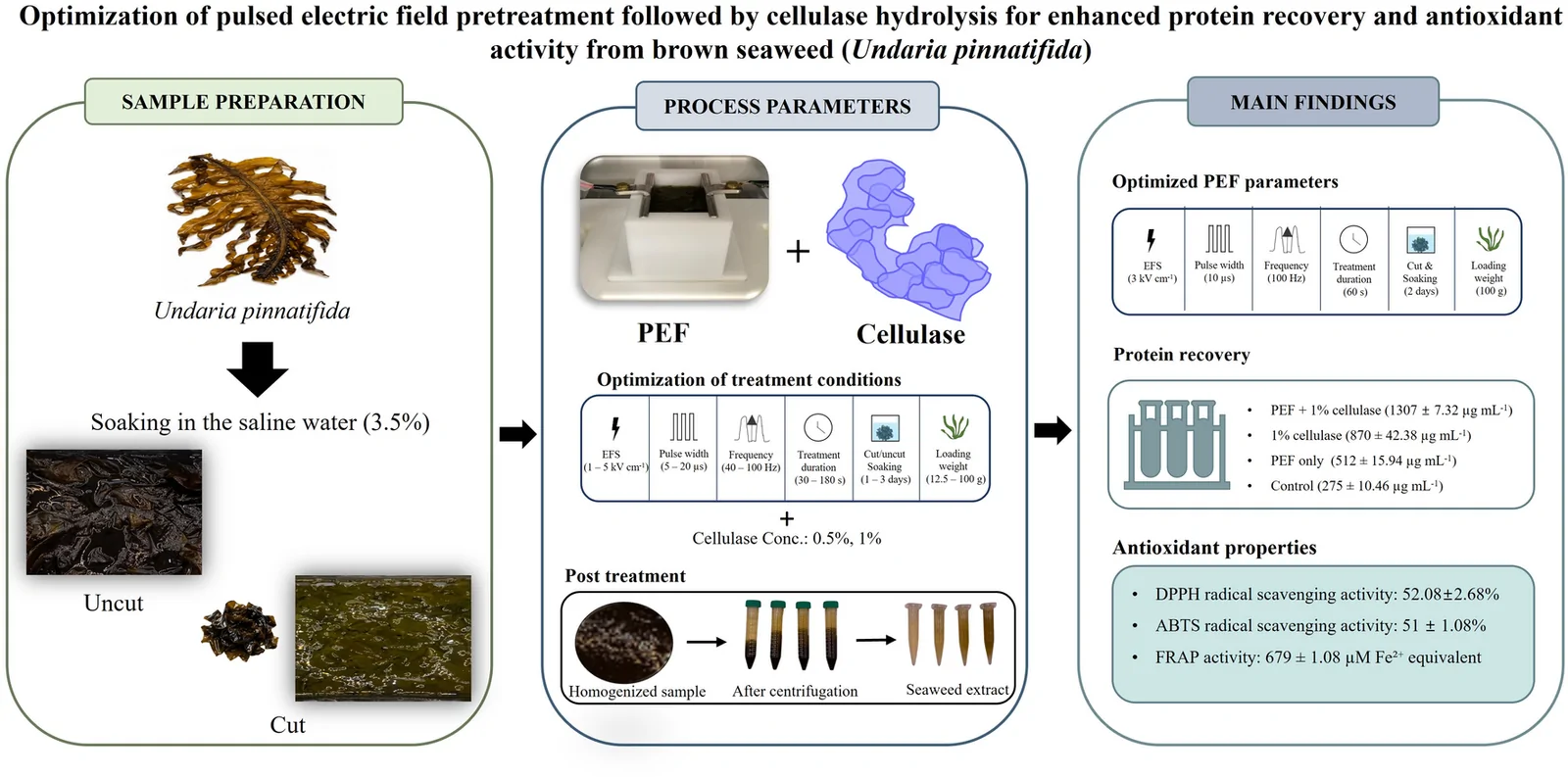

**Supplementary Information:**

The online version contains supplementary material available at https://doi.org/10.1186/s40643-026-01140-2.

## Highlights

Optimal PEF conditions included 3 kV cm^−1^, 10 µs, 100 Hz, and 60 s.

Cutting followed by 2-day soaking in 3.5% saline water yielded 493 ± 2.06 µg mL^−1^ protein after PEF.

Optimized PEF required 0.18 kJ and achieved an energy efficiency of 684.72 ± 2.78 mg protein kJ^−1^.

PEF followed by 1% cellulase increased protein concentration to 1307 ± 38.62 µg mL^−1^.

PEF + cellulase achieved 52.08 ± 2.68% DPPH and 51.00 ± 1.08% ABTS radical-scavenging activity.

PEF + cellulase produced the highest FRAP response at 679 ± 1.08 µM Fe^2+^ equivalents.

**Supplementary Information:**

The online version contains supplementary material available at https://doi.org/10.1186/s40643-026-01140-2.

## Introduction

Marine macroalgae, commonly known as seaweed, have attracted increasing attention as valuable sources of bioactive compounds with applications in food, nutraceuticals, pharmaceuticals, and biotechnology (Park et al. 2024). Among the three major groups of seaweed, brown macroalgae constitute the largest group in terms of thallus size and total biomass and are strongly influenced by climatic conditions (Fleurence and Levine 2016). Compared with green macroalgae, red and brown macroalgae exhibit greater morphological and anatomical differentiation (Lobban and Harrison 1994). Brown seaweeds are particularly rich in protein, dietary fiber, polysaccharides, and antioxidant compounds, making them promising candidates for protein extraction and biorefinery applications (Zhou et al. 2024). In this context, *Undaria pinnatifida* represents a valuable raw material for the recovery of proteins and other high-value bioactive ingredients (Kumar et al. 2021; Ghelichi and Jacobsen 2025). On a dry-weight basis, *U. pinnatifida* contains approximately 10–18% moisture, 11–14% protein, 0.5–2% lipids, and 38–48% carbohydrates, along with a high dietary fiber content (~ 35%), making it a promising biomass for protein recovery and marine biorefinery applications (Fleurence and Levine 2016). Its relatively high protein fraction among brown seaweeds further supports its suitability as a feedstock for extraction and biorefinery processes (Nadeeshani et al. 2022). However, efficient protein extraction from *U. pinnatifida* remains challenging due to the complex structure of its cell wall, which consists of multiple layers of alginates, fucoidan, laminarin, and cellulose that create strong mass transfer resistance and hinder the release of intracellular compounds (Bojorges et al. 2025). Therefore, improving the accessibility of intracellular proteins while maintaining the functional properties of the recovered compounds is a key challenge in the valorization of *U. pinnatifida* biomass.

Conventional extraction methods such as Soxhlet extraction, maceration, decoction, infusion, percolation, and digestion are simple and widely used; however, they typically require long extraction times, high solvent consumption, and, in some cases, elevated temperatures that can degrade thermolabile bioactive compounds (Bitwell et al. 2023; Fonseca-Barahona et al. 2026). Although thermal extraction methods rely on heat transfer via conduction, convection, and radiation, convection-based heating in particular may induce substantial thermal stress, leading to degradation of heat-sensitive bioactive compounds (Picot-Allain et al. 2021). In contrast, non-thermal technologies such as pulsed electric field (PEF) have emerged as promising alternatives for enhancing extraction efficiency while preserving bioactive integrity. PEF induces electroporation in cell membranes, thereby improving mass transfer and facilitating the recovery of bioactive compounds under mild processing conditions (Bashir et al. 2026; Luzi et al. 2026a). Therefore, the development of efficient, non-thermal, and environmentally sustainable extraction technologies is essential for improving protein recovery while maintaining the functional and nutritional quality of seaweed-derived bioactive compounds (Ali et al. 2021). From a process-development perspective, PEF is particularly attractive because its effects can be controlled through operational parameters such as electric field strength, pulse width, pulse repetition frequency, and treatment duration, allowing treatment severity and energy input to be adjusted according to the characteristics of the biomass (Raso et al. 2016).

Pulsed electric field technology has been widely studied as a non-thermal processing technique for enhancing extraction from plant and marine materials (Bocker and Silva 2022; Khojasteh et al. 2025). It involves the application of short, high-voltage electrical pulses (typically 1–40 kV cm^−1^) between electrodes, generating an electric field that induces transient or permanent pores in cellular membranes, a phenomenon known as electroporation (Barba et al. 2017). This membrane permeabilization increases cell membrane conductivity and reduces mass transfer resistance, thereby facilitating the release of intracellular constituents such as proteins, peptides, and antioxidants (Ranjha et al. 2021). PEF has been successfully applied to improve extraction yields of juices, oils, proteins, phenolics, anthocyanins, and other bioactive compounds from fruits, vegetables, meat, fish, and algae, while also reducing energy consumption and minimizing thermal degradation (Steinbruch et al. 2024; Abad et al. 2025; Wang et al. 2026; Zhao et al. 2026). However, PEF primarily affects cellular membranes and may not completely overcome the rigid, polysaccharide-rich cell wall characteristic of brown seaweeds. While PEF effectively permeabilizes cell membranes, it does not fully disrupt the structural barriers formed by cellulose and other cell-wall polysaccharides, which can continue to restrict the release of intracellular proteins (Steinbruch et al. 2023; Maribu et al. 2024). Consequently, optimization of PEF alone may not be sufficient to maximize protein recovery from *U. pinnatifida*, indicating the need for a complementary cell-wall-disruption strategy.

Enzymatic hydrolysis using cell wall-degrading enzymes such as cellulase, alginate lyase, and laminarinase has been shown to improve the release of intracellular compounds by degrading structural polysaccharides (Costa et al. 2021). Nevertheless, enzymatic treatment alone is often limited by restricted enzyme penetration into intact cells and the high structural resistance of seaweed biomass (Steinbruch et al. 2023). Therefore, combining PEF as a physical pretreatment with enzymatic hydrolysis offers a complementary approach: PEF can increase cellular permeability and alter mass-transfer properties, while subsequent enzymatic hydrolysis can degrade accessible cell-wall polysaccharides and potentially improve protein release (Maribu et al. 2024). The sequential application of these treatments is therefore of particular interest because the structural changes produced by PEF may increase the accessibility of cellulose-containing regions to cellulase, while enzymatic hydrolysis can further weaken barriers that remain after electroporation. Despite increasing interest in PEF and enzymatic treatments separately, limited research has investigated their combined effect on protein extraction from *U. pinnatifida*. In addition, optimization of key PEF parameters, including electric field strength, pulse width, pulse repetition frequency, and treatment time, remains insufficiently explored for brown seaweed systems. Moreover, the extent to which optimized PEF pretreatment improves the effectiveness of subsequent cellulase hydrolysis, compared with either treatment applied individually, remains insufficiently characterized. Furthermore, comprehensive evaluation of the antioxidant properties of resulting protein-containing extracts using multiple assays, such as 2,2-diphenyl-1-picrylhydrazyl (DPPH), 2,2′-azino-bis(3-ethylbenzothiazoline-6-sulfonic acid) (ABTS), and ferric reducing antioxidant power (FRAP) assays, remains limited. Together, these gaps highlight the need to evaluate PEF optimization and subsequent enzymatic hydrolysis within an integrated process rather than as independent extraction treatments.

Therefore, the present study investigated an integrated PEF pretreatment–cellulase hydrolysis process for enhanced protein recovery and antioxidant activity from *U. pinnatifida*. The study hypothesized that PEF-induced membrane permeabilization would increase the accessibility of the seaweed matrix to subsequent cellulase hydrolysis, thereby providing greater protein release than either PEF or enzymatic treatment alone. Specifically, this study aimed to (1) optimize PEF treatment parameters, including electric field strength, pulse width, pulse repetition frequency, and treatment duration, to maximize protein release; (2) evaluate the effects of seaweed loading and physical pretreatment conditions, including cutting and saline soaking, on extraction efficiency; (3) characterize the optimized PEF process in terms of protein recovery, electrical conductivity, energy input, and energy efficiency; (4) evaluate the effect of combining optimized PEF pretreatment with cellulase hydrolysis and compare the combined treatment with PEF-only and cellulase-only treatments; and (5) characterize the antioxidant activities of the resulting extracts using DPPH, ABTS, and FRAP assays.

By integrating PEF optimization with subsequent cellulase hydrolysis, this study provides a sequential physical–enzymatic approach to overcoming the structural barriers that limit protein release from *U. pinnatifida*. The combined evaluation of extraction performance, electrical conductivity, energy input, and energy efficiency provides insight into the process characteristics of the optimized PEF treatment, while DPPH, ABTS, and FRAP assays assess the functional antioxidant properties of the resulting extracts. The findings therefore provide a laboratory-scale basis for evaluating PEF-assisted enzymatic processing as a mild and resource-efficient strategy for the recovery of protein and other value-added compounds from brown seaweed biomass.

## Materials and methods

### Materials

Unless otherwise stated, all chemicals and reagents were of analytical grade and were obtained from Sigma-Aldrich (St. Louis, MO, USA). The bicinchoninic acid (BCA) Protein Assay Kit was obtained from Thermo Scientific (Rockford, IL, USA), and cellulase was purchased from Tokyo Chemical Industry Co., Ltd. (Tokyo, Japan). The chemicals used for antioxidant assays included 2,2-diphenyl-1-picrylhydrazyl (DPPH), 2,2′-azino-bis(3-ethylbenzothiazoline-6-sulfonic acid) (ABTS), potassium persulfate, 2,4,6-tris(2-pyridyl)-s-triazine (TPTZ), ferric chloride hexahydrate (FeCl_3_·6 H_2_O), ferrous sulfate heptahydrate (FeSO_4_·7 H_2_O), Trolox, and L-ascorbic acid. Hydrochloric acid (HCl), sodium acetate, sodium chloride, and other reagents were also analytical grade. The commercial cellulase preparation was used as received and added at concentrations of 0.5% and 1.0% (*w/w*, based on substrate mass). The enzyme preparation had a declared activity of 17,000 U g^−1^ according to the manufacturer’s specification.

### Sample preparation

The brown seaweed *Undaria pinnatifida* was purchased from the Jungang Traditional Seafood Market, Tongyeong, Republic of Korea, and transported to the laboratory in an ice box. Upon arrival, the samples were immediately processed to minimize changes in moisture content and biochemical composition during storage. The seaweed was rinsed three times with tap water followed by distilled water to remove adhering sand, salts, epiphytes, and other surface contaminants. After washing, excess surface water was removed, and the cleaned samples were used for subsequent pretreatment and PEF experiments. The cleaned seaweed thalli were manually cut into approximately 1 × 1 cm segments using a stainless-steel knife. For experiments investigating the effect of pretreatment, both cut and uncut seaweed were evaluated. The samples were soaked in 3.5% (*w/v*) sodium chloride (NaCl) solution for 1, 2, or 3 days at ambient temperature, as specified for each experimental condition. The 3.5% NaCl concentration was selected to provide a saline environment comparable to seawater and to facilitate hydration and removal of loosely associated soluble materials from the seaweed matrix (Polikovsky et al. 2016; Solana et al. 2025). After soaking, the samples were drained before PEF treatment, and the same draining procedure was applied to all experimental groups. Unless otherwise stated, the optimized pretreatment consisted of cutting the thallus into approximately 1 × 1 cm pieces followed by 2 days of soaking in 3.5% NaCl solution.

### PEF system and treatment conditions

#### PEF system description

**Fig. 1:**
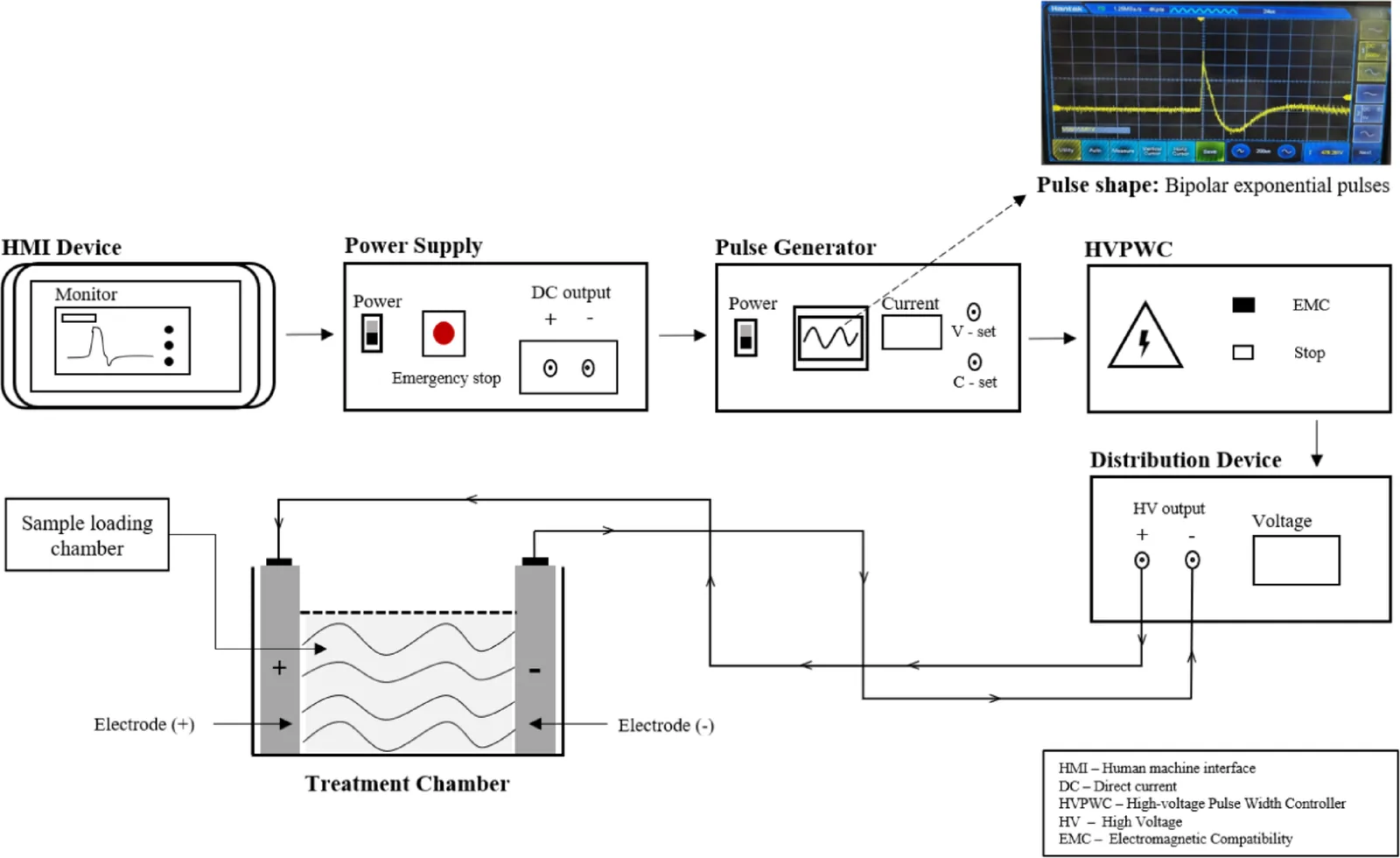
Schematic diagram of a pulsed electric field (PEF) system for marine products

Pulsed electric field (PEF) treatment is a non-thermal processing technology in which short-duration, high-voltage electrical pulses are applied to biological materials to induce electroporation and increase cell or tissue permeability (Bashir et al. 2026). PEF treatments were performed using a magnetron pulse-modulator-based PEF system (PEFCIA-40; Chorusing, Daegu, Republic of Korea), operated through a human–machine interface (HMI) for setting and monitoring the treatment parameters (Fig. 1). The PEF system consisted of a power supply, pulse generator, magnetron pulse modulator, high-voltage electrical assembly, treatment chamber, power distribution unit, and HMI control system. The equipment was capable of generating bipolar exponential pulses over a range of operating conditions. The system specifications included an electric field strength of 1–40 kV cm^−1^, electric current of 1–50 A, pulse width of 5–20 µs, pulse repetition frequency of 40–100 Hz, and a maximum modulator peak power of 4 MW. The treatment chamber used a working volume of 250 mL and was equipped with two opposing parallel-plate electrodes separated by a 6 cm electrode gap. The electric field strength (EFS) was determined from the applied voltage (V) and electrode distance (d) according to EFS = V/d. The treatment chamber was operated at ambient temperature unless otherwise specified. The sample temperature was monitored before and after PEF treatment using a probe-type digital thermometer (FS100 BK, Fuzhou Eclock Electronics Technology Co., Ltd., Fuzhou, China), as repeated electrical pulsing may increase sample temperature and potentially affect protein extraction.

#### PEF treatment procedure

The preliminary PEF treatment condition was set at 3 kV cm^−1^ electric field strength, 10 µs pulse width, 40 Hz pulse repetition frequency, and 60 s treatment duration. These conditions were subsequently modified according to the individual optimization experiments. For each treatment, the designated mass of pretreated *U. pinnatifida* was introduced into the treatment chamber containing 150 mL of distilled water. Unless otherwise specified, the standard biomass loading was 100 g. PEF treatment was then applied using the designated electric field strength, pulse width, pulse repetition frequency, and treatment duration. The number of pulses delivered during each treatment was calculated from pulse repetition frequency and treatment duration (number of pulses = frequency × treatment time). This parameter was used to describe the electrical exposure applied to the sample.

Electrical conductivity (EC) of the sample was measured immediately before and after PEF treatment using an EC meter (MM4-EC; TOA DKK-TOA Co., Tokyo, Japan). Changes in EC were monitored as an indirect indicator of changes in sample electrical properties associated with tissue permeabilization and ion release.

The electrical energy input was calculated according to Eq. (1) (Timmermans et al. 2019).

1 $$ E = U\times I \times t $$

where *E* is the total electrical energy input (J), *U* is the applied voltage (V), *I* is the electric current (A), and *t* is the treatment duration (s).

Specific energy input was calculated by dividing the total electrical energy by the treated sample volume and was expressed as kJ L^−1^. Total energy consumption was also reported as kJ per treatment. The calculated energy was normalized to the total treatment volume (250 mL; 100 g biomass plus 150 mL distilled water under the standard loading condition) and expressed as kJ L^−1^.

Following PEF treatment, the samples were homogenized using a Top^®^ MS-280D laboratory stirrer (MS280D; MTOPS/Misung Scientific Co., Ltd., Yangju-si, Gyeonggi-do, Republic of Korea) at 3000 rpm for 30–60 s. Homogenization was performed on ice to minimize temperature increases during mechanical disruption. The homogenized samples were maintained at 4 °C, and aliquots were collected at 5, 15, 30, 45, 60, 120, and 180 min. Each aliquot was centrifuged at 4000 × g for 15 min at 4 °C using a LABOGENE 1248R refrigerated centrifuge (GYROZEN Co. Ltd., Gimpo, Republic of Korea). The resulting supernatants were collected and used for protein quantification. Unless otherwise stated, three independent experimental replicates were performed for each treatment condition.

### Optimization of PEF processing parameters to enhance protein recovery and process efficiency

A sequential one-factor-at-a-time (OFAT) experimental approach was used to identify PEF conditions that provided high protein release while avoiding unnecessary electrical energy input. One operating parameter was varied at a time while the remaining parameters were maintained at the selected baseline condition. The condition providing the highest protein concentration, while also considering protein recovery and energy-related responses, was selected for the subsequent optimization stage.

#### Electric field strength

Electric field strength is a critical PEF parameter because it determines the magnitude of the induced transmembrane potential and consequently influences the extent of electroporation (Wang et al. 2018). Insufficient field strength may result in limited membrane permeabilization and reduced protein release, while excessive field strength can increase energy consumption and the risk of thermal damage or electrical arcing (Angersbach et al. 2000). To determine the optimal treatment condition, electric field strengths of 1, 2, 3, 4, and 5 kV cm^−1^ were investigated. Pulse width, pulse repetition frequency, and treatment duration were maintained at 10 µs, 40 Hz, and 60 s, respectively. Protein concentration, protein recovery, EC, and energy-related parameters were determined for each treatment condition. The electric field strength providing the most favorable balance between protein recovery and energy input was selected for subsequent experiments.

#### Pulse width

Pulse width determines the duration of each electric pulse and therefore contributes to the cumulative electric exposure of the sample (Taha et al. 2022). Excessively short pulse durations may not provide sufficient energy to induce effective electroporation, whereas prolonged pulses may increase the likelihood of undesirable thermal effects (Assaf et al. 2026). Pulse widths of 5, 10, 15, and 20 µs were evaluated at an electric field strength of 3 kV cm^−1^, pulse repetition frequency of 40 Hz, and treatment duration of 60 s. The influence of pulse width was evaluated using protein concentration and protein recovery as the primary responses, together with EC and energy input as process-related responses.

#### Pulse repetition frequency

Pulse repetition frequency determines the number of pulses delivered per unit time and therefore affects cumulative electrical exposure and energy input (Gorgey and Dudley 2008; García-Sánchez et al. 2018). Increasing the frequency may enhance extraction efficiency through greater membrane permeabilization; however, excessive frequencies can contribute to sample heating (Raso et al. 2016). Pulse repetition frequencies of 40, 60, 80, and 100 Hz were evaluated at 3 kV cm^−1^, 10 µs, and 60 s. The number of pulses delivered at each frequency was calculated as 2400, 3600, 4800, and 6000 pulses, respectively. Protein concentration, protein recovery, EC, and energy efficiency were determined for each condition.

#### Treatment duration

Treatment duration determines the total period during which the biomass is exposed to the electrical field and may influence both electroporation efficiency and mass transfer (Aronsson et al. 2001). Insufficient treatment times may limit protein release, whereas prolonged treatments can increase energy consumption without providing additional extraction benefits (Alirezalu et al. 2020). Treatment durations of 30, 60, 90, 120, 150, and 180 s were evaluated at 3 kV cm^−1^, 10 µs, and 100 Hz. Protein concentration, recovery, EC, and energy efficiency were determined to identify the treatment duration providing the most favorable extraction performance.

#### Seaweed loading weight

Sample loading can affect the effective treatment volume, electrical characteristics of the sample, and mass transfer during PEF processing (Golberg et al. 2016). To assess the effect of biomass, loading weights of 12.5, 25, 50, 75, and 100 g were investigated. PEF treatment was performed at 3 kV cm^−1^, 10 µs, 100 Hz, and 60 s. The effect of biomass loading was assessed based on protein concentration, protein recovery, EC, and energy efficiency.

#### Pretreatment conditions

Pretreatment experiments were conducted to determine whether mechanical size reduction and saline soaking could improve the effectiveness of subsequent PEF treatment. *U. pinnatifida* samples were either left uncut or manually cut into approximately 1 × 1 cm segments. The samples were subsequently soaked in 3.5% (*w/v*) NaCl solution for 1, 2, or 3 days. Cutting could increase the exposed surface area and potentially facilitate mass transfer, whereas saline soaking could soften the seaweed tissue while maintaining conditions compatible with the natural marine environment (Pataro et al. 2017). The experimental conditions were therefore uncut + 1 day, uncut + 2 days, uncut + 3 days, cut + 1 day, cut + 2 days, and cut + 3 days. PEF treatment was maintained at 3 kV cm^−1^, 10 µs, 100 Hz, and 60 s, with a seaweed loading weight of 100 g. Protein concentration and protein recovery were used to identify the most effective pretreatment condition for subsequent PEF-assisted enzymatic hydrolysis.

### PEF-assisted enzymatic hydrolysis

To investigate whether enzymatic hydrolysis could further improve protein release following PEF pretreatment, samples treated under the optimized PEF conditions were subjected to cellulase hydrolysis. Cellulase was added at concentrations of 0.5 and 1.0% (*w/w*, based on substrate weight). The PEF-treated seaweed was suspended in the reaction medium at a 1:10 solid-to-liquid ratio (*w/v*). The reaction medium was adjusted to pH 5.0 using 1 N HCl solution. The enzymatic hydrolysis was conducted at 40 °C and 100 rpm using a shaking incubator (HB-201SF; Hanbaek Scientific Co., Bucheon-si, Gyeonggi-do, Republic of Korea), according to the method reported by Postma et al. (2018). Samples were collected at 5, 15, 30, 45, 60, 120, and 180 min. The enzymatic reaction was terminated by heating at 100 °C for 10 min. Samples were then cooled to room temperature and centrifuged at 4000 ×g for 15 min. The supernatants were collected for protein analysis. To distinguish the effects of the individual and combined treatments, the experimental groups included: (i) untreated control, (ii) PEF-only treatment, (iii) cellulase-only treatment, and (iv) PEF + cellulase treatment. Where applicable, the same incubation time and reaction conditions were applied to the corresponding control groups.

### Protein quantification

Protein concentration was quantified using the bicinchoninic acid (BCA) assay according to Bashir et al. (2024). Bovine serum albumin (BSA) provided in the BCA Protein Assay Kit (Thermo Scientific, Rockford, USA) was used as the calibration standard. Briefly, 25 µL of each sample was mixed with 200 µL of freshly prepared working reagent in a 96-well microplate. The plate was incubated at 37 °C for 30 min, after which absorbance was measured at 562 nm using a SPECTROstar Nano microplate reader (BMG LABTECH GmbH, Ortenberg, Germany). A standard curve was generated using the measured absorbance values of the BSA standards, and sample protein concentrations were calculated from the resulting regression equation. Protein concentration was expressed as µg mL^−1^ extract.

### Calculation of protein recovery and energy efficiency

Protein recovery was calculated as the percentage of the initial protein content in the seaweed biomass recovered in the extract, according to Eq. (2).

2 $$ {\mathrm{Protein~recovery}}\left( {{\% }} \right) = \frac{{M_{{{\mathrm{extracted}}}} }}{{M_{{{\mathrm{initial}}}} }}{\mathrm{~}} \times 100 $$

here *M*_extracted_ was calculated from the measured protein concentration (mg) and the total volume of the recovered extract, whereas *M*_initial_ was determined from the initial protein content (mg) of the corresponding seaweed biomass.

Importantly, protein concentration (µg mL^−1^) and protein recovery (%) were treated as separate response variables. Protein concentration describes the concentration of protein in the recovered liquid extract, whereas protein recovery accounts for the total protein recovered relative to the initial protein content of the biomass.

To evaluate the energy performance of the PEF process, energy efficiency was calculated as the amount of protein recovered per unit energy consumed, according to Eq. (3).

3 $$ {\mathrm{Energy~efficiency}} = \frac{{M_{{{\mathrm{recovered}}}} }}{{E_{{{\mathrm{input}}}} }} $$

where *M*_recovered_ is the mass of recovered protein (mg) and *E*_input_ is the electrical energy consumed during PEF treatment (kJ). Energy efficiency was expressed as mg protein kJ^−1^.

### Antioxidant activity assays

#### DPPHradical-scavenging activity

DPPH radical-scavenging activity was determined using a modified method based on Bashir et al. (2020). 2,2-Diphenyl-1-picrylhydrazyl (DPPH) was prepared at a concentration of 100 µM in methanol. L-ascorbic acid (10 mg mL^−1^) was used as a positive control. Briefly, 50 µL of sample extract was mixed with 250 µL of DPPH solution in a 96-well microplate. The reaction mixture was incubated in the dark at room temperature for 30 min. Absorbance was subsequently measured at 517 nm using the SPECTROstar Nano microplate reader. DPPH radical-scavenging activity was calculated according to Eq. (4) and expressed as the percentage inhibition of DPPH radical.

4 $$ \begin{aligned} & {\mathrm{DPPH}} - {\mathrm{radical~scavenging~activity~}}\left( {{\% }} \right) \\ & = \frac{{A_{{{\mathrm{control}}}} - A_{{{\mathrm{sample}}}} }}{{A_{{{\mathrm{control}}}} }}{\mathrm{~}} \times 100 \\ \end{aligned}$$

where *A*_control_ is the absorbance of the DPPH solution without sample and *A*_sample_ is the absorbance of the reaction mixture containing the sample. In addition, sample blank correction was performed to minimize interference from the seaweed extracts, as their intrinsic color can interfere with spectrophotometric measurements of antioxidant activity.

#### ABTS radical-scavenging activity

ABTS radical-scavenging activity was determined according to a modified method of Bashir et al. (2018). The ABTS radical cation was generated by reacting 7 mM 2,2′-azino-bis(3-ethylbenzothiazoline-6-sulfonic acid) (ABTS) with 2.45 mM potassium persulfate and incubating the mixture in the dark overnight. The resulting solution was diluted with distilled water to an absorbance of 0.70 ± 0.02 at 734 nm. Briefly, 50 µL of sample extract was mixed with 250 µL of ABTS working solution and incubated in the dark at room temperature for 10 min. Absorbance was measured at 734 nm using a microplate reader (BMG LABTECH GmbH). Trolox (10 mg mL^− 1^) was used as a positive control. ABTS radical scavenging activity was calculated according to Eq. (5) and expressed as the percentage inhibition of ABTS radical.

5 $$ {\mathrm{ABTS~inhibition~}}\left( {{\% }} \right) = \frac{{A_{{{\mathrm{control}}}} - A_{{{\mathrm{sample}}}} }}{{A_{{{\mathrm{control}}}} }}{\mathrm{~}} \times 100 $$

where *A*_control_ is the absorbance of the ABTS working solution without sample and *A*_sample_ is the absorbance in the presence of the sample. In addition, sample blanks were included to correct for the intrinsic absorbance of seaweed extracts.

#### Ferric reducing antioxidant power (FRAP)

Ferric reducing antioxidant power (FRAP) was determined according to Benzie and Strain (1996). The FRAP reagent consisted of 300 mM sodium acetate buffer (pH 3.6), 10 mM 2,4,6-tris(2-pyridyl)-s-triazine (TPTZ) prepared in 40 mM hydrochloric acid, and 20 mM ferric chloride (FeCl_3_·6 H_2_O), mixed at a 10:1:1 (*v/v/v*) ratio. The reagent was pre-warmed at 37 °C for 10 min. A 50 µL aliquot of sample was mixed with 150 µL of FRAP reagent and incubated at room temperature for 8 min. Absorbance was measured at 593 nm using a microplate reader (BMG LABTECH GmbH). Ferrous sulfate heptahydrate (FeSO_4_·7 H_2_O) was used as the calibration standard, and FRAP values were expressed as µM Fe^2+^ equivalents.

### Statistical analysis

All experiments were conducted using three independent experimental replicates, unless otherwise stated. For statistical analysis, the mean of the three technical measurements from each independent experimental replicate was used as the experimental observation (*n* = 3). Results are presented as mean ± standard deviation (SD). Statistical analyses were performed using IBM SPSS Statistics version 27 (IBM Corp., Armonk, NY, USA). Levene’s test was used to assess homogeneity of variances before conducting ANOVA. For experiments involving a single independent factor, such as electric field strength, pulse width, pulse repetition frequency, or treatment duration, one-way analysis of variance (ANOVA) followed by Tukey’s honestly significant difference (HSD) post-hoc test was used to evaluate differences among treatment means. For experiments involving two independent factors, such as PEF treatment × biomass loading and cutting condition × saline-soaking duration, two-way ANOVA was performed to evaluate the main effects and interaction effects. Significant interactions were further examined using appropriate post-hoc multiple-comparison procedures. An independent-samples Student’s *t*-test was used only for comparisons involving two independent treatment groups. Pearson correlation analysis was performed only when a predefined relationship between continuous variables was evaluated. Statistical significance was defined as *p* < 0.05.

## Results and discussion

### Optimization of PEF process parameters for protein release

Pulsed electric field (PEF) treatment can enhance the release of intracellular compounds by increasing cell membrane permeability through electroporation (Tomasevic et al. 2023; Chudasama et al. 2025). The effectiveness of PEF depends on the applied electric field strength and pulse characteristics, including pulse width, repetition frequency, and treatment duration (Raso et al. 2016; Luzi et al. 2026b). This is particularly relevant to *U. pinnatifida*, whose structurally complex, polysaccharide-rich matrix can restrict protein release (Echave et al. 2021; Pedro et al. 2021). Therefore, these four PEF parameters were systematically evaluated to identify the conditions that provided the highest protein release under the experimental conditions used in this study.

#### Effect of electric field strength on protein release

**Fig. 2:**
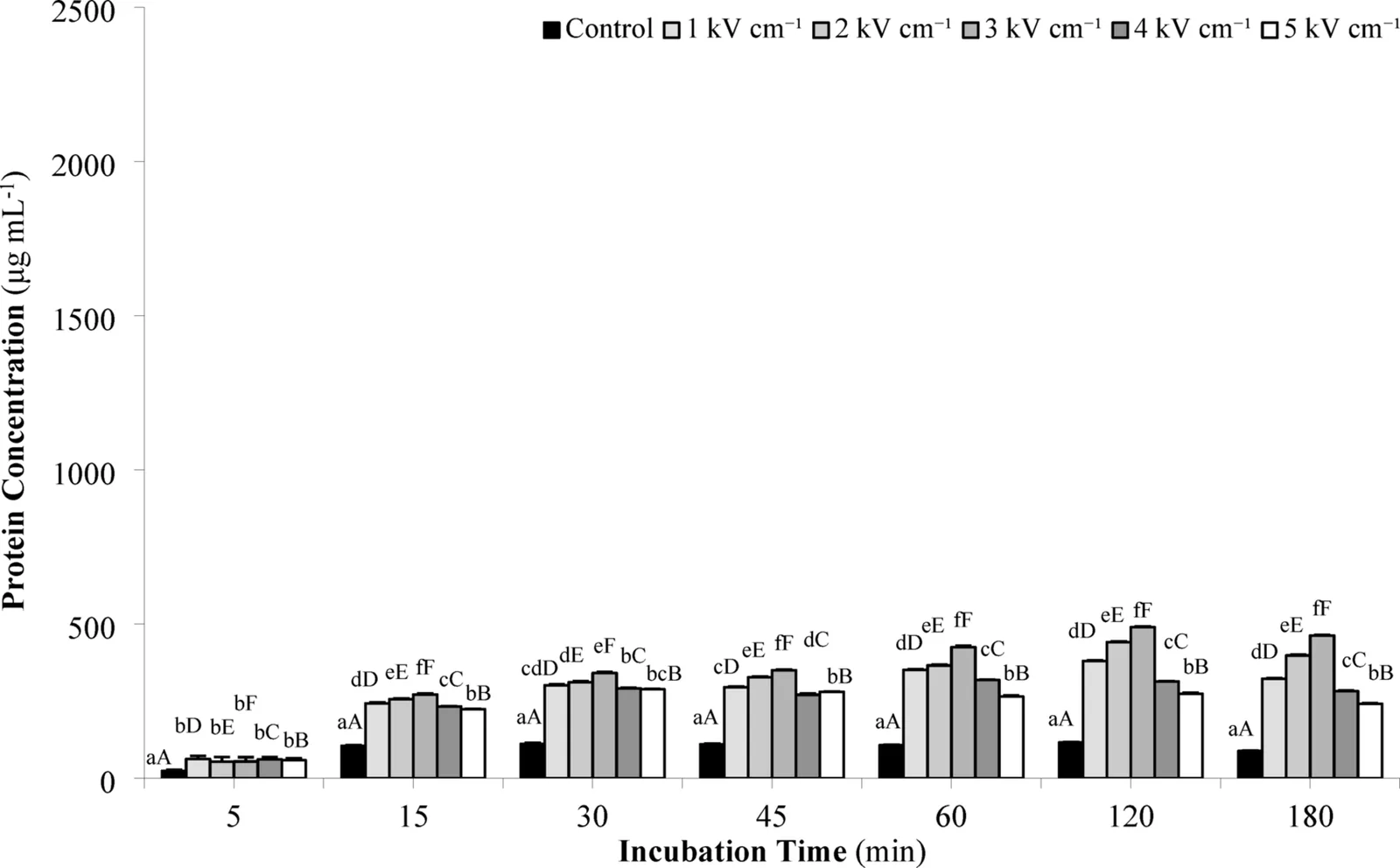
Effect of applied electric field strength on protein release from *Undaria pinnatifida* during PEF treatment. Values are expressed as mean ± SD (*n* = 3). Different uppercase superscript letters (A–F) indicate significant differences among treatments, whereas different lowercase superscript letters (a–f) indicate significant differences among incubation times within the same treatment. Statistical significance was determined using Tukey’s post-hoc test (F[5, 12] = 4592.60, *p* < 0.001)

Electric field strength had a pronounced effect on protein release from *U. pinnatifida* (Fig. 2). At the evaluated conditions, protein concentration increased from 116 ± 3.61 µg mL^−1^ in the untreated control to 380 ± 2.00, 442 ± 2.36, and 490 ± 3.23 µg mL^−1^ at 1, 2, and 3 kV cm^−1^, respectively, after 120 min of post-treatment incubation. Increasing the field strength further, however, reduced the protein concentration to 314 ± 1.53 µg mL^−1^ at 4 kV cm^−1^ and 274 ± 3.51 µg mL^−1^ at 5 kV cm^−1^. Thus, the response was clearly non-linear, with 3 kV cm^−1^ producing the highest protein concentration within the investigated range. Statistical analysis using IBM SPSS Statistics version 27 confirmed a significant effect of electric field strength on protein concentration (F[5, 12] = 4592.60, *p* < 0.001). Tukey’s multiple-comparison test showed that the 3 kV cm^−1^ treatment was significantly different from 1, 2, 4, and 5 kV cm^−1^ treatments, as well as from the control (Fig. 2). Similar non-linear extraction responses have been reported for PEF-assisted extraction, where the final extraction efficiency depends on the balance between membrane permeabilization, treatment severity, energy input, and the characteristics of the biological matrix (Gonzalez and Barrett 2010; Vaessen et al. 2019).

The increase in protein concentration from 116 to 490 µg mL^−1^ represents an approximately 3.2-fold increase relative to the untreated control, demonstrating a substantial improvement in protein release at the optimum field strength. However, the subsequent decline at 4 and 5 kV cm^−1^ demonstrates that increasing field strength beyond 3 kV cm^−1^ did not further improve protein recovery. This behavior was consistent with the electroporation mechanism of PEF, whereby an applied electric field induces changes in cell membrane permeability and facilitates the migration of intracellular compounds into the surrounding medium (Tomasevic et al. 2023; Chudasama et al. 2025). However, in the present macroalgal system, the decrease at higher field strengths may reflect excessive treatment severity and/or non-beneficial energy dissipation rather than insufficient electroporation. Because the present experiments did not directly measure cell-wall damage, or individual membrane-pore characteristics, these mechanisms should be regarded as possible explanations rather than experimentally demonstrated causes. Accordingly, 3 kV cm^−1^ was selected as the optimum field strength within the tested range. Importantly, this selection was based on the highest experimentally measured protein concentration and its statistical distinction from the other treatments, rather than on the assumption that a higher electric field would necessarily provide better extraction.

#### Influence of PEF pulse width on protein release

**Fig. 3:**
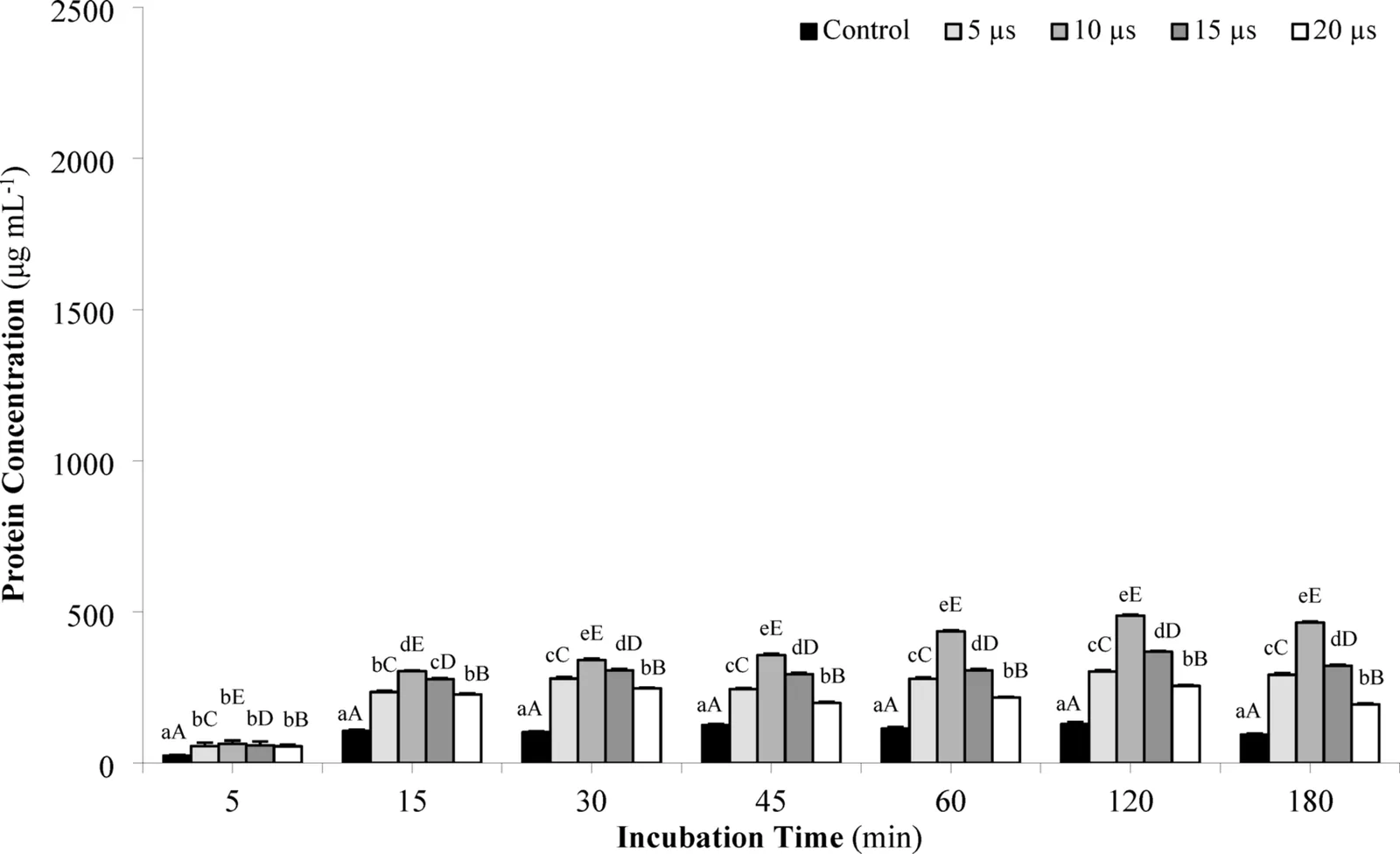
Impact of pulse width on protein release from *Undaria pinnatifida* during PEF treatment. Values are expressed as mean ± SD (*n* = 3). Different uppercase superscript letters (A–E) indicate significant differences among treatments, whereas different lowercase superscript letters (a–e) indicate significant differences among incubation times within the same treatment. Statistical significance was determined using Tukey’s post-hoc test (F[4, 10] = 17,988.66, *p* < 0.001)

After selecting 3 kV cm^−1^, the influence of pulse width was investigated (Fig. 3). Protein concentration increased from 130 ± 5.50 µg mL^−1^ in the untreated control to 303 ± 4.51 µg mL^−1^ at 5 µs and reached a maximum of 487 ± 3.54 µg mL^−1^ at 10 µs. Increasing the pulse width to 15 and 20 µs resulted in lower concentrations of 369 ± 2.40 and 255 ± 3.51 µg mL^−1^, respectively. Therefore, the response to pulse width was also non-linear, with 10 µs providing the highest protein release. The statistical analysis showed a significant effect of pulse width (F(4, 10] = 17988.66, *p* < 0.001). Tukey’s multiple-comparison test further indicated that the 10 µs treatment resulted in significantly higher protein concentration than the 5, 15, and 20 µs treatments, as well as the control.

The protein concentration obtained at 10 µs was approximately 2.7-fold higher than that of the untreated control, indicating that this pulse width provided the most effective extraction response among the conditions evaluated. Importantly, extending the pulse beyond this condition did not produce a corresponding increase in protein recovery. This finding is particularly relevant to process optimization because it demonstrates that pulse duration should not be optimized independently of treatment severity. Pulse width determines the duration over which electrical energy is delivered during each pulse and therefore contributes to the overall electrical exposure of the biomass (Taha et al. 2022). The reduction in protein concentration at 15 and 20 µs suggests that extending pulse duration beyond the optimum did not improve the extraction process under the present conditions. Previous PEF studies have similarly demonstrated that extraction efficiency does not necessarily increase continuously with increasing pulse duration because excessive electrical exposure can increase energy consumption without producing proportional improvements in mass transfer (Li et al. 2021). Thus, the present results identify 10 µs as an experimentally supported operating window for *U. pinnatifida* protein extraction at 3 kV cm^−1^.

#### Effect of pulse repetition frequency on protein release

**Fig. 4:**
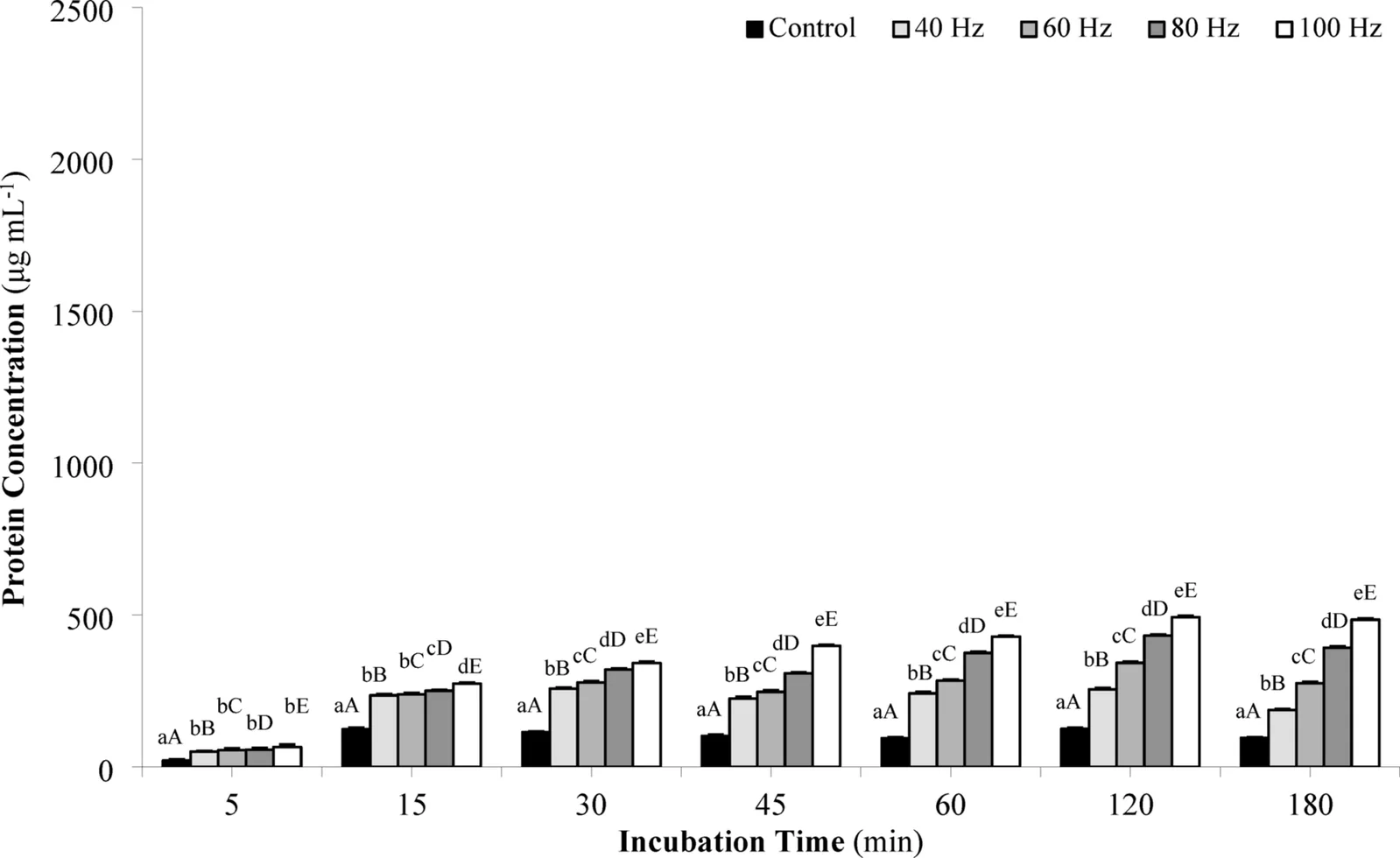
Effect of pulse repetition frequency on protein release from *Undaria pinnatifida* during PEF treatment. Values are expressed as mean ± SD (*n* = 3). Different uppercase superscript letters (A–E) indicate significant differences among treatments, whereas different lowercase superscript letters (a–e) indicate significant differences among incubation times within the same treatment. Statistical significance was determined using Tukey’s post-hoc test (F[24, 60] = 425.571, *p* < 0.001)

Pulse repetition frequency was subsequently evaluated at the selected field strength and pulse width (Fig. 4). Protein concentration increased progressively from 255 ± 5.28 µg mL^−1^ at 40 Hz to 343 ± 4.73 µg mL^−1^ at 60 Hz, 432 ± 3.52 µg mL^−1^ at 80 Hz, and 493 ± 3.52 µg mL^−1^ at 100 Hz, compared with 125 ± 4.51 µg mL^−1^ in the untreated control. Statistical analysis using two-way ANOVA revealed significant effects of pulse repetition frequency, post-treatment incubation time, and their interaction on protein concentration (frequency: F[4, 60] = 8466.36, *p* < 0.001; incubation time: F[6, 60] = 6083.018, *p* < 0.001; frequency × incubation time: F[24, 60] = 425.571, *p* < 0.001). Unlike electric field strength and pulse width, for which the response declined within the tested range after an optimum was reached, protein release increased continuously from 40 to 100 Hz, showing a 2.9-fold increase at 100 Hz. This suggests that, under the present conditions, increasing the pulse repetition frequency delivered during the fixed treatment period enhanced the extent of PEF-induced permeabilization, thereby enhancing protein release. The increasing response may be associated with the greater number of electrical pulses applied to the biomass, resulting in more frequent electroporation events and greater cumulative permeabilization. This interpretation is consistent with previous PEF studies showing that pulse number and repetition frequency can influence treatment severity and mass transfer (Ayub and Fincan 2023; Lytras et al. 2024). Nevertheless, the present study investigated frequencies only up to 100 Hz. Therefore, 100 Hz should be interpreted as the optimum within the investigated experimental range, rather than as evidence that frequencies above 100 Hz would necessarily reduce extraction efficiency. Based on the highest measured protein concentration, 100 Hz was selected for the subsequent treatment-duration experiment.

#### Effect of PEF treatment duration on protein recovery

**Fig. 5:**
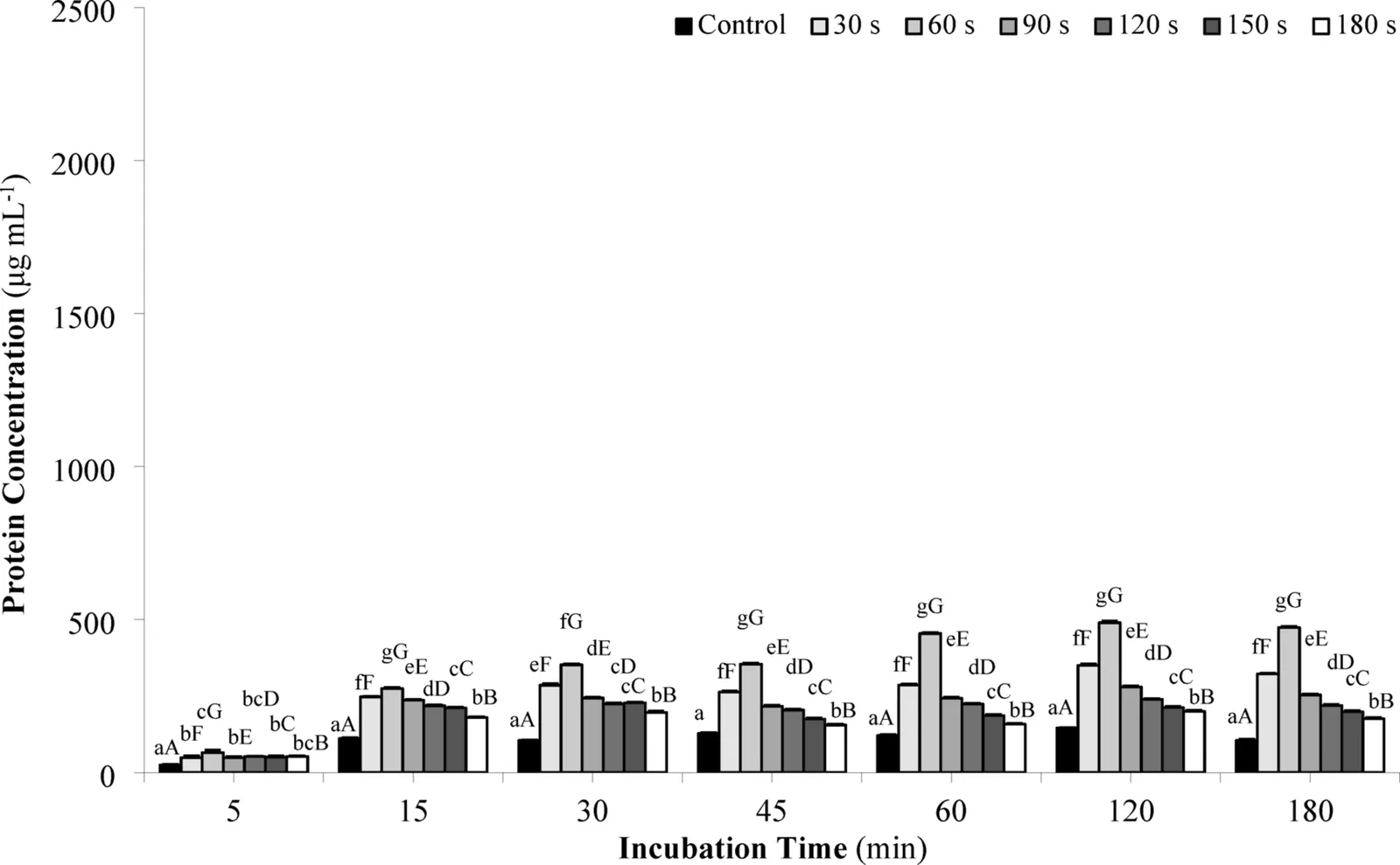
Impact of PEF treatment duration on protein release from *Undaria pinnatifida* during PEF treatment. Values are expressed as mean ± SD (*n* = 3). Different uppercase superscript letters (A–G) indicate significant differences among treatments, whereas different lowercase superscript letters (a–g) indicate significant differences among incubation times within the same treatment. Statistical significance was determined using Tukey’s post-hoc test (F[6, 14] = 8739.83, *p* < 0.001)

Treatment duration produced another clear optimum in protein release (Fig. 5). Protein concentration increased from 146 ± 1.73 µg mL^−1^ in the untreated control to 350 ± 5.16 µg mL^−1^ after 30 s and reached a maximum of 490 ± 5.14 µg mL^−1^ after 60 s. Further extension of the treatment reduced protein concentration to 280 ± 4.02, 240 ± 2.17, 212 ± 4.16, and 200 ± 3.61 µg mL^−1^ at 90, 120, 150, and 180 s, respectively. Statistical analysis demonstrated a significant effect of treatment duration (F[6,14] = 8739.83, *p* < 0.001). Tukey’s HSD test showed that the 60 s treatment produced significantly higher protein concentration than the other treatment durations, whereas protein concentration decreased significantly at longer treatment durations.

The concentration at 60 s was approximately 2.35-fold higher than the untreated control, demonstrating the substantial effect of treatment duration on protein release. The maximum protein release after 60 s indicates that extending the electrical treatment beyond this duration did not improve protein extraction under the selected field strength, pulse width, and frequency. Instead, the progressive reduction after 60 s suggests that longer exposure was not advantageous for net protein recovery. From a process-engineering perspective, this result is important because prolonged electrical treatment increases treatment time and electrical energy input without necessarily increasing extract recovery. Comparable optimization behavior has been reported for PEF-assisted extraction, where treatment beyond an effective permeabilization window does not necessarily result in proportional increases in extraction yield (Raso et al. 2016). Therefore, 60 s was selected as the optimum treatment duration, providing the highest experimentally measured protein concentration within the tested range.

Overall, the four electrical-parameter experiments established 3 kV cm^−1^, 10 µs, 100 Hz, and 60 s as the optimum PEF conditions within the investigated ranges. The different response patterns observed for the individual parameters also demonstrate why optimization based solely on increasing treatment intensity would be inappropriate for this biomass.

### Effect of biomass loading and pretreatment conditions

Following optimization of the electrical parameters, the influence of biomass-related variables was evaluated because the efficiency of PEF treatment is also dependent on the physical characteristics and concentration of the material introduced into the treatment chamber.

#### Effect of seaweed loading weight on protein concentration

The effect of seaweed loading was evaluated using the optimized electrical conditions of 3 kV cm^−1^, 10 µs, 100 Hz, and 60 s (Table 1). Protein concentration increased with increasing biomass loading, from 115 ± 4.54 µg mL^−1^ at 12.5 g to 217 ± 3.13, 280 ± 2.00, 411 ± 3.52, and 491 ± 4.00 µg mL^−1^ at 25, 50, 75, and 100 g, respectively. The corresponding untreated controls exhibited protein concentrations of 84 ± 3.03, 172 ± 2.65, 138 ± 3.52, 189 ± 2.08, and 205 ± 3.90 µg mL^−1^, respectively. Among the tested biomass loads, the 100 g treatment yielded the highest protein concentration. Statistical analysis revealed significant effects of biomass loading and the PEF treatment × biomass loading interaction (loading: F[4, 20] = 10,441.90, *p* < 0.001; PEF × loading: F[4, 20] = 1,707.93, *p* < 0.001). The continuous increase up to 100 g indicates that, within the experimental configuration used, increasing biomass loading provided greater protein recovery. However, biomass loading cannot be considered independently of treatment geometry. The present experiments were conducted in a fixed-volume treatment chamber, meaning that changes in loading may alter biomass-to-liquid ratio, electric-field distribution, electrode contact, and mass-transfer conditions. Consequently, 100 g represents the optimum biomass loading for the present experimental configuration rather than a universal optimum for *U. pinnatifida* extraction. This qualification is important for scale-up because a loading that performs well in a laboratory batch chamber may not provide the same field distribution or energy efficiency in a larger or continuous PEF system (Golberg et al. 2016). Thus, the result provides an experimentally defined operating condition for the subsequent experiments rather than a general biomass-loading recommendation.

**Table 1:** Effect of pulsed electric field (PEF) treatment and seaweed loading concentration on protein content

Sample	Seaweed (g)	Protein concentration (µg mL^−1^) at different incubation time (min)						
		5	15	30	45	60	120	180
Control	12.5	14 ± 1.53^Aa^	84 ± 3.03^Aa^	67 ± 2.04^Aa^	72 ± 1.66^Aa^	42 ± 1.85^Aa^	55 ± 1.79^Aa^	41 ± 2.52^Aa^
PEF		34 ± 2.89^Bc^	112 ± 1.79^Bb^	115 ± 4.54^Bb^	110 ± 3.35^Bb^	94 ± 4.22^Bb^	104 ± 4.46^Bb^	83 ± 2.36^Bb^
Control	25	16 ± 2.52^Cab^	138 ± 3.52^Cc^	121 ± 2.54^Cb^	126 ± 2.32^Cc^	120 ± 3.79^Cc^	134 ± 1.93^Cc^	99 ± 3.01^Cc^
PEF		37 ± 3.51^Fd^	192 ± 1.93^Ff^	184 ± 2.82^Fe^	178 ± 5.22^Ff^	186 ± 2.72^Fe^	217 ± 3.13^Fg^	182 ± 2.49^Ff^
Control	50	19 ± 3.06^Dab^	154 ± 3.00^Dd^	149 ± 5.16^Dc^	145 ± 4.24^Dd^	156 ± 3.21^Dd^	172 ± 2.65^Dd^	128 ± 3.03^Dd^
PEF		40 ± 4.16^Gd^	217 ± 2.52^Gh^	235 ± 3.61^Gg^	254 ± 3.06^Gg^	238 ± 2.99^Gg^	280 ± 2.00^Gh^	258 ± 2.50^Gh^
Control	75	24 ± 1.00^Eabc^	164 ± 3.51^Ee^	173 ± 2.04^Ed^	159 ± 4.14^Ee^	189 ± 2.08^Ee^	183 ± 3.07^Ee^	145 ± 3.76^Ee^
PEF		53 ± 3.51^He^	243 ± 3.99^Hi^	302 ± 2.08^Hh^	287 ± 3.51^Hh^	334 ± 3.09^Hh^	411 ± 3.52^Hi^	391 ± 3.00^Hi^
Control	100	25 ± 2.52^Ic^	205 ± 3.90^Ig^	197 ± 4.04^If^	178 ± 1.45^If^	200 ± 3.39^If^	204 ± 2.01^If^	190 ± 1.92^Ig^
PEF		65 ± 8.19^Jf^	276 ± 3.79^Jj^	375 ± 3.61^Jj^	386 ± 4.73^Ji^	425 ± 3.51^Ji^	491 ± 4.00^Jj^	466 ± 4.58^Jj^

Values are expressed as mean ± SD (*n* = 3). PEF: Pulsed electric field. Statistical analyses were performed using one-way and two-way ANOVA, followed by Tukey’s multiple-comparison test. Significant differences (*p* < 0.05) were observed in protein concentration as a function of seaweed sample weight and incubation time (F[4, 20] = 1707.93, *p* < 0.001). Different uppercase superscript letters (A–J) indicate significant differences among treatment groups, whereas different lowercase superscript letters (a–j) indicate significant differences among incubation times within the same treatment

#### Effect of pretreatment conditions

Mechanical cutting and saline soaking were then evaluated to determine whether modification of the seaweed matrix before PEF could improve protein release (Table 2). Among the tested conditions, mechanically cut seaweed followed by 2 days of soaking in 3.5% saline solution produced the highest protein concentration, 493 ± 2.06 µg mL^−1^. The corresponding values after 1 and 3 days of soaking were 444 ± 4.04 and 403 ± 2.28 µg mL^−1^, respectively. For uncut seaweed, protein concentrations were 337 ± 2.19, 363 ± 4.08, and 310 ± 2.44 µg mL^−1^ after 1, 2, and 3 days of soaking, respectively. Statistical analysis using two-way ANOVA demonstrated a significant main effect of cutting condition (F[1, 30] = 141.03, *p* < 0.001) and soaking duration (F[2, 30] = 4.86, *p* = 0.015), whereas the cutting × soaking-duration interaction was not significant (F[2, 30] = 0.34, *p* = 0.715). Tukey’s HSD test was subsequently used for appropriate pairwise comparisons among treatment means. The highest protein concentration was obtained from cut seaweed soaked for 2 days. Cutting increases exposed surface area and decreases the characteristic distance over which soluble components must migrate, potentially improving hydration and subsequent mass transfer (O’Connor et al. 2020; Solana et al. 2025).

**Table 2:** Impact of seaweed sample preparation and soaking period on protein recovery following pulsed electric field (PEF) treatment

Sample	Pretreatment conditions	Protein concentration (µg mL^−1^) at different incubation time (min)						
		5	15	30	45	60	120	180
Control	Uncut with 1 day soaking	23 ± 1.53^B^	127 ± 2.71^Bb^	173 ± 2.05^Bb^	150 ± 2.90^Bb^	155 ± 3.51^Bb^	184 ± 2.41^Bb^	153 ± 4.16^Bb^
PEF		23 ± 1.53^H^	262 ± 4.07^Hg^	334 ± 4.00^Hg^	314 ± 2.49^Hh^	337 ± 2.19^Hg^	335 ± 4.01^Hh^	308 ± 2.33^Hg^
Control	Cut with 1 day soaking	23 ± 2.52^E^	174 ± 2.63^Ed^	220 ± 3.55^Ed^	208 ± 3.81^Ee^	225 ± 4.14^Ee^	237 ± 1.82^Ee^	194 ± 3.21^Ed^
PEF		25 ± 1.00^K^	342 ± 3.04^Kj^	405 ± 3.61^Kj^	401 ± 3.62^Kk^	410 ± 3.54^Kj^	444 ± 4.04^Kk^	405 ± 4.14^Kj^
Control	Uncut with 2 days soaking	23 ± 1.53^C^	126 ± 3.68^Cb^	191 ± 3.01^Cc^	174 ± 3.68^Cc^	185 ± 3.09^Cc^	203 ± 2.11^Cc^	171 ± 2.00^Cc^
PEF		25 ± 1.53^I^	282 ± 2.50^Ih^	363 ± 4.08^Ih^	344 ± 2.29^Ii^	352 ± 1.84^Ih^	356 ± 4.58^Ii^	320 ± 2.62^Ih^
Control	Cut with 2 days soaking	23 ± 2.52^F^	195 ± 3.29^Fe^	249 ± 1.96^Fe^	220 ± 1.49^Ff^	234 ± 2.87^Fe^	261 ± 4.60^Ff^	222 ± 2.54^Fe^
PEF		25 ± 2.00^L^	434 ± 3.00^Lk^	459 ± 4.36^Lk^	466 ± 3.21^Ll^	446 ± 4.16^Lk^	493 ± 2.06^Ll^	463 ± 2.18^Lk^
Control	Uncut with 3 days soaking	23 ± 1.53^A^	105 ± 3.00^Aa^	157 ± 3.63^Aa^	115 ± 3.94^Aa^	121 ± 1.85^Aa^	153 ± 3.70^Aa^	125 ± 3.73^Aa^
PEF		24 ± 1.53^G^	239 ± 3.48^Gf^	303 ± 2.38^Gf^	271 ± 3.28^Gg^	310 ± 2.44^Gf^	297 ± 3.27^Gg^	263 ± 2.83^Gg^
Control	Cut with 3 days soaking	22 ± 2.08^D^	153 ± 2.08^Dc^	199 ± 3.62^Dc^	191 ± 2.54^Dd^	201 ± 2.10^Dd^	215 ± 3.20^Dd^	188 ± 2.32^Dd^
PEF		24 ± 2.52^J^	297 ± 3.00^Ji^	383 ± 2.00^Ji^	371 ± 1.32^Jj^	389 ± 3.58^Ji^	403 ± 2.28^Jj^	385 ± 3.15^Ji^

Values are expressed as mean ± SD (*n* = 3). PEF, Pulsed electric field. Statistical analyses were performed using one-way and two-way ANOVA, followed by Tukey’s multiple-comparison test. Significant differences (*p* < 0.05) were observed in protein concentration as a function of pretreatment condition and incubation time (F[2, 30] = 0.34, *p* = 0.715). Different uppercase superscript letters (A–L) indicate significant differences among treatment groups, whereas different lowercase superscript letters (a–l) indicate significant differences among pretreatment conditions within the same treatment

The soaking period also affected protein recovery. The increase from 1 to 2 days suggests that controlled hydration and tissue softening improved the subsequent extraction process. In contrast, extending soaking to 3 days reduced protein concentration, indicating that prolonged hydration did not provide an additional extraction advantage under the present conditions. Therefore, the response was not simply proportional to soaking duration. The combination of mechanical cutting and 2 days of soaking in 3.5% saline solution was consequently selected as the optimum pretreatment. These findings demonstrate that PEF efficiency depended not only on electrical parameters but also on the physical state of the biomass entering the treatment chamber. This is particularly relevant for macroalgae because their structurally complex, polysaccharide-rich matrices can restrict solvent penetration and intracellular mass transfer (Echave et al. 2021; Pedro et al. 2021).

### Performance of the optimized PEF treatment

The optimized treatment consisted of 3 kV cm^−1^ electric field strength, 10 µs pulse width, 100 Hz pulse repetition frequency, 60 s treatment duration, 100 g biomass loading, and mechanical cutting followed by 2 days of soaking in 3.5% saline solution. Under these conditions, the protein concentration reached 493 ± 2.06 µg mL^−1^ after 120 min of post-treatment incubation (Table 3). Based on the initial protein content of the corresponding seaweed biomass and the total protein mass recovered in the extract, the calculated protein recovery was 5.98 ± 0.02%. The increase in electrical conductivity from 708 ± 5.29 to 1350 ± 4.00 µS cm^−1^ following PEF treatment provides indirect evidence of altered electrical properties and increased ionic mobility in the treated biomass. The increase in conductivity is consistent with increased ionic mobility and release of intracellular soluble components following membrane disruption (Toepfl et al. 2007; Ghoshal 2023). Nevertheless, conductivity is an indirect indicator of membrane permeabilization and cannot by itself demonstrate protein-specific release (Raso et al. 2016). Because electrical conductivity is influenced by ionic concentration, temperature, and matrix composition, the observed increase should be interpreted as a supportive process indicator rather than direct evidence of membrane permeabilization.

**Table 3:** Effect of pulsed electric field (PEF) treatment on the electrical conductivity of *Undaria pinnatifida* throughout incubation

Incubation period (min)	Electrical conductivity (µs cm^−1^)	
	Control	PEF
Before treatment	344 ± 4.00^aA^	357 ± 7.02^aB^
After treatment	–	877 ± 12.70^bB^
5	708 ± 5.29^fA^	1350 ± 4.00^hB^
15	641 ± 5.03^eA^	1244 ± 4.00^gB^
30	621 ± 5.03^cA^	1145 ± 3.06^fB^
45	667 ± 5.09^dA^	1149 ± 9.02^fB^
60	667 ± 6.11^dA^	1089 ± 3.06^eB^
120	626 ± 8.72^cA^	987 ± 5.03^dB^
180	607 ± 5.03^bA^	889 ± 5.03^cB^

Values are expressed as mean ± SD (*n* = 3). PEF: Pulsed electric field. Statistical analyses were performed using one-way and two-way ANOVA, followed by Tukey’s multiple-comparison test. Significant differences (*p* < 0.05) were observed in electrical conductivity between measurements taken before and after treatment. Different uppercase superscript letters (A, B) indicate significant differences between treatment groups, whereas different lowercase superscript letters (a–h) indicate significant differences among incubation times within the same treatment

The measured temperature remained within 23.1–23.5 °C, indicating that the optimized process produced only a minimal temperature change. This observation supports the interpretation that the increase in protein release was predominantly associated with electrical treatment rather than substantial bulk heating. This is consistent with the principal advantage of PEF as a non-thermal or mild-thermal processing technology (Pliquett 2003; Meneses et al. 2011; Astráin-Redín et al. 2022).

The total electrical energy input was 0.18 kJ, corresponding to a specific energy input of 0.72 kJ L^−1^. The resulting energy efficiency was 684.72 ± 2.78 mg protein kJ^−1^. The low measured energy input under the laboratory conditions is encouraging for the use of PEF as a pretreatment. However, this energy performance should not be interpreted as a direct prediction of industrial energy efficiency because electrode configuration, electrical losses, treatment volume, biomass loading, and continuous-processing conditions can substantially alter energy consumption (Golberg et al. 2016). Therefore, the present values represent laboratory-scale energy performance under the specified treatment configuration rather than industrial-scale energy efficiency.

### Effect of cellulase-assisted hydrolysis following PEF pretreatment

**Fig. 6:**
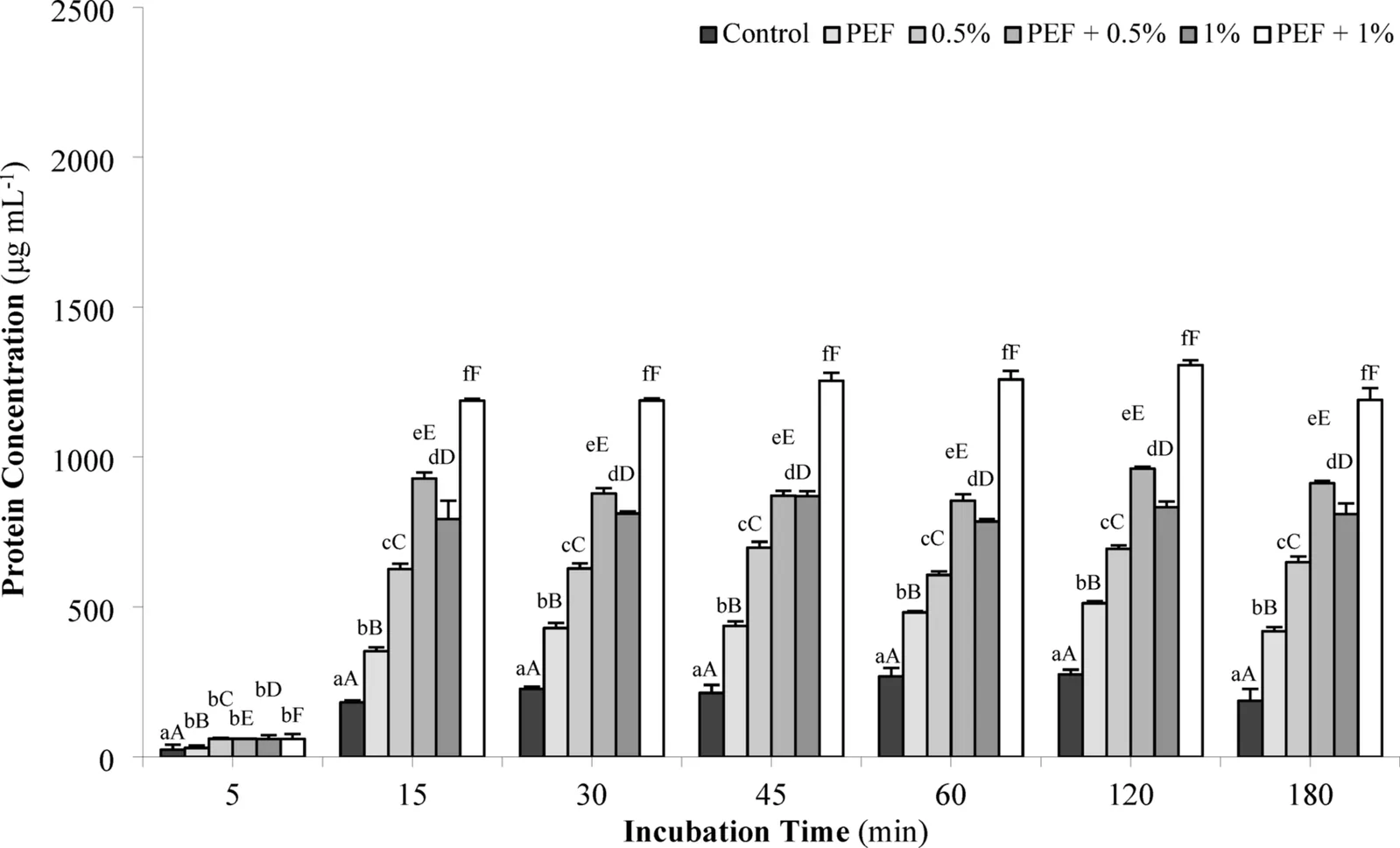
Combined effect of pulsed electric field (PEF) and enzymolysis on protein extraction from *Undaria pinnatifida*. Values are expressed as mean ± SD (*n* = 3). Different uppercase superscript letters (A–F) indicate significant differences among treatment groups, whereas different lowercase superscript letters (a–f) indicate significant differences among incubation times. Statistical significance was determined using Tukey’s post-hoc test (F[1, 14] = 0.71, *p* = 0.412)

The optimized PEF treatment was subsequently combined with cellulase-assisted hydrolysis to determine whether enzymatic degradation of the seaweed structural matrix could further improve protein release (Fig. 6). The PEF + 1% cellulase treatment produced the highest protein concentration (1307 ± 38.62 µg mL^−1^), followed by PEF + 0.5% cellulase (961 ± 30.11 µg mL^−1^). In comparison, 1% and 0.5% cellulase alone produced 870 ± 42.38 and 698 ± 40.81 µg mL^−1^, respectively, whereas PEF alone and the untreated control produced 512 ± 15.94 and 275 ± 10.46 µg mL^−1^, respectively. Thus, the PEF + 1% cellulase treatment produced approximately 50% more protein than 1% cellulase alone, 155% more than PEF alone, and 375% more than the untreated control. Importantly, the combined treatment also produced a higher protein concentration than 1% cellulase alone, demonstrating that PEF pretreatment enhanced the subsequent cellulase-assisted release of protein from the seaweed matrix. Two-way ANOVA demonstrated significant main effects of PEF treatment and cellulase concentration on protein concentration (PEF: F[1, 14] = 20.51, *p* < 0.001; cellulase concentration: F[1, 14] = 83.69, *p* < 0.001), whereas the PEF × cellulase interaction was not significant (F[1, 14] = 0.71, *p* = 0.412). These results indicate that both PEF treatment and cellulase concentration independently contributed to the observed increase in protein concentration, although the statistical analysis did not demonstrate a significant interaction between the two factors. Nevertheless, the numerical protein concentration obtained with PEF + 1% cellulase was higher than that obtained with 1% cellulase alone, supporting the practical benefit of applying PEF before cellulase hydrolysis under the conditions investigated. PEF increases cellular permeability and disrupts structural integrity, potentially improving access of cellulase to the polysaccharide-rich matrix, while cellulase hydrolyzes cellulose-containing structural regions that remain after PEF treatment (Steinbruch et al. 2023; Maribu et al. 2024). The present data therefore provide direct experimental support for using optimized PEF as a pretreatment before enzymatic hydrolysis. The increase from 512 ± 15.94 µg mL^−1^ with PEF alone to 1307 ± 38.62 µg mL^−1^ with PEF + 1% cellulase is particularly important because it demonstrates that the principal benefit of the integrated process was not merely the effect of PEF itself but the improved effectiveness of the subsequent enzymatic step.

### Antioxidant activities of the recovered extract

**Fig. 7:**
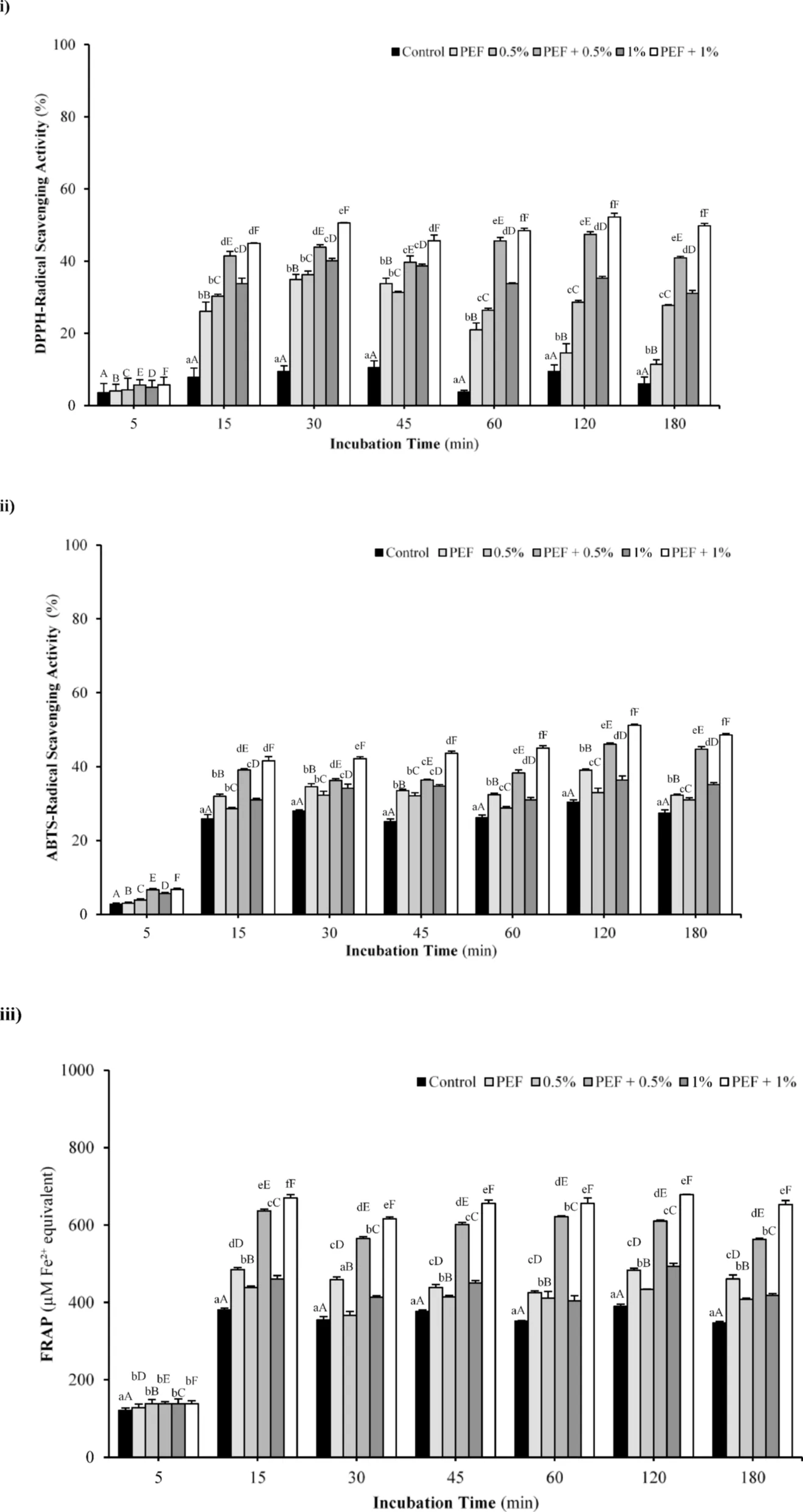
Antioxidant activities of *Undaria pinnatifida* protein extracts following PEF-assisted enzymolysis. (i) DPPH radical-scavenging activity, (ii) ABTS-radial scavenging activity, and (iii) FRAP activity. Values are expressed as mean ± SD (*n* = 3). Different uppercase superscript letters (A–F) indicate significant differences among treatment groups, whereas different lowercase superscript letters (a–f) indicate significant differences among incubation times, according to Tukey’s post-hoc test (*p* < 0.05)

The increase in protein extraction following the PEF–cellulase treatment was accompanied by changes in the antioxidant properties of the recovered extracts (Fig. 7). Antioxidant activity was evaluated separately using three complementary assays: 2,2-diphenyl-1-picrylhydrazyl (DPPH) radical-scavenging activity, 2,2′-azinobis-(3-ethylbenzothiazoline-6-sulfonic acid) (ABTS) radical-scavenging activity, and ferric reducing antioxidant power (FRAP). Each assay was conducted independently to characterize a different aspect of antioxidant behavior. The results obtained from the three assays were subsequently compared across the extraction treatments to determine whether the observed treatment effects were consistent across different measures of antioxidant activity.

#### DPPH radical scavenging activity

DPPH radical-scavenging activity was highest for PEF + 1% cellulase (52.08 ± 2.68%), followed by PEF + 0.5% cellulase (47.35 ± 1.17%) (Fig. 7i). The corresponding activities were 40.95 ± 1.31% for 1% cellulase, 36.04 ± 0.96% for 0.5% cellulase, 34.91 ± 1.40% for PEF alone, and 10.49 ± 1.91% for untreated control. The PEF + 1% cellulase extract therefore exhibited approximately four-fold higher DPPH-scavenging activity than the untreated control. Statistical analysis revealed a significant effect of treatment (F[5, 12] = 36,711.11, *p* < 0.001). Tukey’s multiple-comparison test indicated the following order of DPPH-scavenging activity: PEF + 1% cellulase > PEF + 0.5% cellulase > 1% cellulase > 0.5% cellulase > PEF > control. The higher activity following the combined treatment indicates that the integrated process increased the radical-scavenging capacity of the recovered extract. This may reflect improved liberation of antioxidant constituents from the seaweed matrix. Brown seaweeds contain diverse compounds with antioxidant potential, including phlorotannins, pigments, peptides, and polysaccharide-associated components (Fonseca-Barahona et al. 2025). However, because the present study did not identify or quantify individual antioxidant compounds, the observed DPPH response should be attributed to increased antioxidant activity of the extract, rather than to a specific compound.

#### ABTS radical-scavenging activity

A similar treatment ranking was observed for ABTS radical-scavenging activity (Fig. 7ii). The PEF + 1% cellulase treatment produced 51.00 ± 1.08% activity, followed by PEF + 0.5% cellulase at 46.00 ± 0.44%. The corresponding values for 1% cellulase, 0.5% cellulase, PEF alone, and the untreated control were 36.00 ± 1.53%, 33.00 ± 1.00%, 39.00 ± 0.38%, and 30.00 ± 0.64%, respectively. Statistical analysis revealed a significant effect of treatment (F[5, 12] = 587.37, *p* < 0.001). Tukey’s HSD test indicated the following treatment ranking for ABTS radical-scavenging activity: PEF + 1% cellulase > PEF + 0.5% cellulase > PEF > 1% cellulase > 0.5% cellulase > control, subject to confirmation of the pairwise significance results from the post-hoc analysis. The greater ABTS response after combined PEF–cellulase treatment, together with the DPPH results, indicates that the integrated treatment increased the overall radical-scavenging capacity of the recovered extract. Importantly, the agreement between DPPH and ABTS strengthens the interpretation because the two assays do not represent identical chemical reactions. Nevertheless, neither assay identifies which specific antioxidant constituents are responsible for the observed activity.

#### Ferric reducing antioxidant power

The FRAP assay provided an additional assessment of the antioxidant capacity of the extracts (Fig. 7iii). The highest value was again obtained with PEF + 1% cellulase (679 ± 1.08 µM Fe^2+^ equivalents), followed by PEF + 0.5% cellulase (636 ± 5.0 µM Fe^2+^ equivalents). The values for 1% cellulase, 0.5% cellulase, PEF alone, and the untreated control were 494 ± 7.1, 434 ± 3.6, 483 ± 5.5, and 390 ± 5.7 µM Fe²⁺ equivalents, respectively. The PEF + 1% cellulase treatment therefore produced approximately a 74% higher FRAP value than the untreated control. Statistical analysis revealed a significant effect of treatment (F[5, 12] = 4819.23, *p* < 0.001). Tukey’s HSD test showed that the PEF + 1% cellulase treatment produced significantly higher FRAP value than the other treatments (*p* < 0.05). The consistent superiority of the PEF + 1% cellulase extract across DPPH, ABTS, and FRAP provides convergent evidence that the integrated treatment improved the antioxidant functionality of the recovered extract. DPPH and ABTS primarily assess radical-scavenging capacity, whereas FRAP measures ferric-ion reducing capacity; therefore, the agreement across the three assays suggests that the observed improvement was not restricted to a single analytical response. However, these assays measure functional antioxidant activity rather than the concentration of individual antioxidant molecules. Consequently, the present results demonstrate improved antioxidant functionality of the extracts but do not establish which individual compounds were preferentially released.

Taken together, the results demonstrate that protein extraction from *U. pinnatifida* was strongly dependent on both PEF treatment severity and the accessibility of the seaweed matrix. The sequential optimization of electric field strength, pulse width, pulse repetition frequency, treatment duration, biomass loading, and pretreatment conditions established a defined processing window within the experimental ranges investigated. Importantly, the observed responses were not consistently improved by increasing treatment intensity or duration, demonstrating that effective PEF-assisted extraction requires an appropriate balance between membrane permeabilization and treatment severity rather than simply maximizing the applied electrical input (Perez et al. 2026). The increase in protein release together with the increase in electrical conductivity and the minimal change in temperature further supports the interpretation that PEF enhanced mass transfer primarily through permeabilization-associated effects rather than substantial thermal treatment (Marín-Sánchez et al. 2024). These findings are consistent with previous reports describing the importance of treatment severity and process conditions in PEF-assisted extraction (Gonzalez and Barrett 2010; Raso et al. 2016; Vaessen et al. 2019).

The subsequent cellulase experiment provided further evidence for the value of using optimized PEF as a pretreatment rather than as a stand-alone extraction step. The substantially higher protein concentration obtained with PEF followed by cellulase compared with either treatment alone indicates that the two processing stages may act on complementary structural barriers within the seaweed matrix. PEF can facilitate cellular permeabilization and mass transfer, whereas cellulase can further weaken cellulose-containing structural components and improve accessibility of matrix-associated proteins (Steinbruch et al. 2023). The antioxidant responses obtained from the separately conducted DPPH, ABTS, and FRAP assays followed the same overall treatment trend as protein extraction, indicating that the integrated PEF–cellulase treatment improved the measured antioxidant properties of the recovered extracts. However, these assays represent independent measures based on different chemical principles and therefore should be interpreted as complementary evidence rather than as a direct measurement of a single antioxidant property. Furthermore, because individual antioxidant compounds were not identified in the present study, the observed improvements cannot be attributed to specific molecular constituents without further compositional analysis. DPPH and ABTS assays assess radical-scavenging capacity through different reaction mechanisms, including hydrogen-atom transfer and electron-transfer contributions, whereas FRAP primarily evaluates reducing capacity through electron donation (Benzie and Strain 1996; Dudonné et al. 2009). The consistent treatment trend across these independent assays therefore supports an improvement in the measured antioxidant functionality of the extracts, although it does not establish which specific compounds were responsible for this effect.

The principal contribution of the present work is therefore the systematic optimization of PEF processing for *U. pinnatifida* and the subsequent application of the optimized treatment as a defined pretreatment for cellulase-assisted extraction. The results provide experimental support for a sequential physical–enzymatic approach to overcoming the structural limitations of macroalgal biomass. This interpretation is consistent with the findings of Steinbruch et al. (2023), who reported improved extraction performance using combined physical and enzymatic treatment approaches. However, the optimum conditions and measured energy performance identified in the present study are specific to the biomass, treatment configuration, pretreatment conditions, and experimental scale used here and should not be directly extrapolated to industrial processing. Future work should evaluate the optimized process under larger-scale and continuous-flow conditions, including electrode configuration, electric-field homogeneity, biomass-to-liquid ratio, electrical losses, enzyme consumption, and downstream recovery. In addition, detailed compositional characterization of the recovered protein and antioxidant fractions would be necessary to determine which bioactive components are preferentially released and to further establish the functional and biorefinery potential of the process.

## Conclusion

This study established an optimized sequential PEF–cellulase process for improving protein recovery and the measured antioxidant functionality of extracts from *U. pinnatifida*. Within the experimental ranges investigated, the optimum PEF conditions were 3 kV cm^−1^ electric field strength, 10 µs pulse width, 100 Hz pulse repetition frequency, and 60 s treatment duration, combined with 100 g seaweed loading and mechanical cutting followed by 2 days of soaking in 3.5% saline solution. Under these conditions, the optimized PEF treatment produced 493 ± 2.06 µg mL^−1^ protein, and the calculated protein recovery was 5.98 ± 0.02%. PEF treatment increased electrical conductivity from 708 ± 5.29 to 1350 ± 4.00 µS cm^−1^, providing indirect evidence of changes in the electrical properties and ionic mobility of the treated matrix. The total electrical energy input was 0.18 kJ, corresponding to an energy efficiency of 684.72 ± 2.78 mg protein kJ^−1^. More importantly, subsequent cellulase hydrolysis substantially increased protein release. Treatment with 1% cellulase after optimized PEF produced 1307 ± 38.62 µg mL^−1^ protein, compared with 870 ± 42.38 µg mL^−1^ for 1% cellulase alone, 512 ± 15.94 µg mL^−1^ for PEF alone, and 275 ± 10.46 µg mL^−1^ for the untreated control. The combined treatment also produced the highest responses in the separately conducted antioxidant assays, with DPPH radical-scavenging activity of 52.08 ± 2.68%, ABTS radical-scavenging activity of 51.00 ± 1.08%, and FRAP activity of 679 ± 1.08 µM Fe^2+^ equivalents. Collectively, these results demonstrate that optimized PEF pretreatment followed by cellulase hydrolysis substantially enhanced protein extraction and increased the measured antioxidant responses of the recovered extracts compared with the corresponding individual treatments under the conditions investigated.

The findings support the practical benefit of applying PEF as a pretreatment before cellulase hydrolysis under the conditions evaluated. However, the process was evaluated under laboratory-scale batch conditions, and the reported energy performance should not be directly extrapolated to industrial processing. Further studies should therefore assess scale-up and continuous-flow operation, enzyme activity and cost, extract composition, and the recovery of additional valuable seaweed components to determine the broader biorefinery potential of the process.

## Supplementary Information

Below is the link to the electronic supplementary material.

Supplementary Material 1

## Data Availability

The data reporting the findings of this study are contained in this article; further queries could be made to the corresponding author(s).

## Abbreviations

PEF: Pulsed electric field
HMI: Human machine interface
EC: Electrical conductivity
BCA: Bicinchoninic acid
DPPH: 2,2-diphenyl-1-picrylhydrazyl
ABTS: 2,2'-azino-bis (3-ethylbenzothiazoline-6-sulfonic acid)
FRAP: Ferric reducing antioxidant power
LED: Light-emitting diode
BSA: Bovine serum albumin
TPTZ: 2,4,6-Tris(2-pyridyl)-s-triazine
SD: Standard deviation
SPSS: Statistical package for the social sciences/IBM SPSS statistics

## References

[CR1] Abad V, Ruíz A, Grasa J, Calvo B, Escursell N, Peiro T, Raso J, Cebrián G, Álvarez-Lanzarote I (2025) Effect of electrical conductivity on the inactivation of *Anisakis* spp. by PEF. Food Control 179:111571. 10.1016/j.foodcont.2025.111571

[CR2] Ali A, Wei S, Liu Z, Fan X, Sun Q, Xia Q, Liu S, Hao J, Deng C (2021) Non-thermal processing technologies for the recovery of bioactive compounds from marine by-products. LWT-Food Sci Technol 147:111549. 10.1016/j.lwt.2021.111549

[CR3] Alirezalu K, Munekata PES, Parniakov O, Barba FJ, Witt J, Toepfl S, Wiktor A, Lorenzo JM (2020) Pulsed elethe ctric field and mild heating for milk processing: a review on recent advances. J Sci Food Agric 100(1):16–24. 10.1002/jsfa.9942 31328265

[CR4] Angersbach A, Heinz V, Knorr D (2000) Effects of pulsed electric fields on cell membranes in real food systems. Innov Food Sci Emerg Technol 1(2):135–149. 10.1016/S1466-8564(00)00010-2

[CR5] Aronsson K, Lindgren M, Johansson BR, Rönner U (2001) Inactivation of microorganisms using pulsed electric fields: the influence of process parameters on* Escherichia coli*,* Listeria innocua*,* Leuconostoc mesenteroides* and* Saccharomyces cerevisiae*. Innov Food Sci Emerg Technol 2(1):41–54. 10.1016/S1466-8564(01)00021-2

[CR6] Assaf N, Chiavaro E, Rinaldi M (2026) Pulsed electric field effects on plant matrices: a comprehensive review of structural and textural responses. Food Biosci 77:108402. 10.1016/j.fbio.2026.108402

[CR7] Astráin-Redín L, Moya J, Alejandre M, Beitia E, Raso J, Calvo B, Cebrián G, Álvarez I (2022) Improving the microbial inactivation uniformity of pulsed electric field ohmic heating treatments of solid products. LWT-Food Sci Technol 154:112709. 10.1016/j.lwt.2021.112709

[CR8] Ayub M, Fincan M (2023) Visualization of the frequency effect of pulsed electric field on enzymatic browning of peel ground tissue. Food Bioprod Process 142:82–93. 10.1016/j.fbp.2023.09.003

[CR9] Barba FJ, Koubaa M, do Prado-Silva L, Orlien V, Sant’Ana ADS (2017) Mild processing applied to the inactivation of the main foodborne bacterial pathogens: a review. Trends Food Sci Technol 66:20–35. 10.1016/j.tifs.2017.05.011

[CR10] Bashir KMI, Mohibbullah M, An JH, Choi JY, Hong YK, Sohn JH, Kim JS, Choi JS (2018) In vivo antioxidant activity of mackerel (*Scomber japonicus*) muscle protein hydrolysate. PeerJ 6:e6181. 10.7717/peerj.6181 30595992 PMC6305115

[CR11] Bashir KMI, Sohn JH, Kim J, Choi J (2020) Identification and characterization of novel antioxidant peptides from mackerel (*Scomber japonicus*) muscle protein hydrolysates. Food Chem 323:126809. 10.1016/j.foodchem.2020.126809 32330643

[CR12] Bashir KMI, Chakniramol S, Mansoor S, Jahn A, Cho MG, Choi JS (2024) Antioxidant activity of protein hydrolysates from redlip mullet (*Chelon haematocheilus*) muscle and byproducts. Foods 13(18):3009. 10.3390/foods13183009 39335938 PMC11431201

[CR13] Bashir KMI, An HR, Mansoor S, Shin HY, Luzi G, Sohn JH, Choi JS (2026) Innovative applications of pulsed electric field technology in marine biomass processing. Food Bioprocess Tech 19:19. 10.1007/s11947-025-04119-7

[CR14] Benzie IF, Strain JJ (1996) The ferric reducing ability of plasma (FRAP) as a measure of antioxidant power: the FRAP assay. Anal Biochem 239(1):70–76. 10.1006/abio 8660627

[CR15] Bitwell C, Indra SS, Luke C, Kakoma MK (2023) A review of modern and conventional extraction techniques and their applications for extracting phytochemicals from plants. Sci Afr 19:e01585. 10.1016/j.sciaf.2023.e01585

[CR16] Bocker R, Silva EK (2022) Pulsed electric field assisted extraction of natural food pigments and colorings from plant matrices. Food Chem X 15:100398. 10.1016/j.fochx.2022.100398 36211728 PMC9532718

[CR17] Bojorges H, Martínez-Sanz M, Fontes-Candia C, Díaz-Piñero L, Martínez-Abad A, López-Rubio A, Fabra MJ (2025) Protein extraction from the industrial solid residue of brown seaweed after alginate extraction. Food Hydrocoll 159:110708. 10.1016/j.foodhyd.2024.110708

[CR18] Chudasama M, Singh DK, Pradhan RC (2025) Review on electroporation mechanisms for PEF-assisted extraction and microbial inactivation. Food Eng Rev 17:706–726. 10.1007/s12393-025-09416-7

[CR19] Costa M, Pio L, Bule P, Cardoso V, Alfaia CM, Coelho D, Brás J, Fontes CMGA, Prates JAM (2021) An individual alginate lyase is effective in the disruption of *Laminaria digitata* recalcitrant cell wall. Sci Rep 11(1):9706. 10.1038/s41598-021-89278-1 33958695 PMC8102539

[CR20] Dudonné S, Vitrac X, Coutière P, Woillez M, Mérillon JM (2009) Comparative study of antioxidant properties and total phenolic content of 30 plant extracts of industrial interest using DPPH, ABTS, FRAP, SOD, and ORAC assays. J Agric Food Chem 57(5):1768–1774. 10.1021/jf803011r 19199445

[CR21] Echave J, Fraga-Corral M, Garcia-Perez P, Popović-Djordjević J, H Avdović E, Radulović M, Xiao J, Prieto A, Simal-Gandara M J (2021) Seaweed protein hydrolysates and bioactive peptides: extraction, purification, and applications. Mar Drugs 19(9):500. 10.3390/md19090500 34564162 PMC8471739

[CR22] Fleurence J, Levine I (eds) (2016) Seaweed in health and disease prevention. Academic, London

[CR23] Fonseca-Barahona I, Shahbaz K, Baroutian S (2025) Bioactives from brown algae: antioxidant, anti-Inflammatory, anticancer, and antimicrobial potential. Chem Bio Eng Rev 12:e70007. 10.1002/cben.70007

[CR24] Fonseca-Barahona I, Shahbaz K, Baroutian S (2026) Emerging methods to extract bioactive compounds from brown algae: a comprehensive review of performance, readiness, and sustainability. Sep Purif Technol 395:137731. 10.1016/j.seppur.2026.137731

[CR25] García-Sánchez T, Merla C, Fontaine J, Muscat A, Mir LM (2018) Sine wave electropermeabilization reveals the frequency-dependent response of the biological membranes. Biochim Biophys Acta Biomembr 1860(5):1022–1034. 10.1016/j.bbamem.2018.01.018 29410049

[CR26] Ghelichi S, Jacobsen C (2025) Seaweed proteins: properties, extraction, challenges, and prospects. J Food Sci 90:e70418. 10.1111/1750-3841.70418 40686495 PMC12278354

[CR27] Ghoshal G (2023) Comprehensive review on pulsed electric field in food preservation: gaps in current studies for potential future research. Heliyon 9(6):e17532. 10.1016/j.heliyon.2023.e17532 37408918 PMC10318501

[CR28] Golberg A, Sack M, Teissie J, Pataro G, Pliquett U, Saulis G, Stefan T, Miklavcic D, Vorobiev E, Frey W (2016) Energy-efficient biomass processing with pulsed electric fields for bioeconomy and sustainable development. Biotechnol Biofuels 9:94. 10.1186/s13068-016-0508-z 27127539 PMC4848877

[CR29] Gonzalez ME, Barrett DM (2010) Thermal, high pressure, and electric field processing effects on plant cell membrane integrity and relevance to fruit and vegetable quality. J Food Sci 75:R121–R130. 10.1111/j.1750-3841.2010.01763.x 21535564 PMC2995313

[CR30] Gorgey AS, Dudley GA (2008) The role of pulse duration and stimulation duration in maximizing the normalized torque during neuromuscular electrical stimulation. J Orthop Sports Phys Ther 38(8):508–516. https://www.jospt.org/doi/18678958 10.2519/jospt.2008.2734 PMC2554670

[CR31] Khojasteh SK, Elmizadeh A, Sarraf M, Dodange S (2025) Non-thermal innovations in solid food processing: eco-friendly alternatives to thermal methods. Appl Food Res 5(2):101256. 10.1016/j.afres.2025.101256

[CR32] Kumar A, Konar A, Garg S, Kaul SC, Wadhwa R (2021) Experimental evidence and mechanism of action of some popular neuro-nutraceutical herbs. Neurochem Int 149:105124. 10.1016/j.neuint.2021.105124 34245808

[CR33] Li L, Yang R, Zhao W (2021) The effect of pulsed electric fields (PEF) combined with temperature and natural preservatives on the quality and microbiological shelf-life of cantaloupe juice. Foods 10(11):2606. 10.3390/foods10112606 34828887 PMC8622698

[CR34] Lobban CS, Harrison PJ (eds) (1994) Seaweed ecology and physiology. Cambridge University Press, Cambridge

[CR35] Luzi G, Bashir KMI, Lyu W, Jahn A, Cho M-G, Choi J-S (2026a) Numerical modeling of pulsed electric field–enhanced release of active compounds from viscoelastic matrices. J Food Eng 423:113268. 10.1016/j.jfoodeng.2026.113268

[CR36] Luzi G, Bashir KMI, Lyu W, Cho MG, Choi JS (2026b) Mathematical modeling and computational approaches for pulsed electric field processing in food preservation: a comprehensive review. Foods 15:164. 10.3390/foods15010164 41517230 PMC12785869

[CR37] Lytras F, Psakis G, Gatt R, Cebrián G, Raso J, Valdramidis V (2024) Exploring the efficacy of pulsed electric fields (PEF) in microbial inactivation during food processing: a deep dive into the microbial cellular and molecular mechanisms. Innov Food Sci Emerg Technol 95:103732. 10.1016/j.ifset.2024.103732

[CR38] Maribu I, Blikra MJ, Eilertsen KE, Elvevold K (2024) Protein enrichment of the red macroalga *Palmaria palmata* using pulsed electric field and enzymatic processing. J Appl Phycol 36:3665–3673. 10.1007/s10811-024-03338-3 39713085 PMC11659382

[CR39] Marín-Sánchez J, Berzosa A, Álvarez I, Sánchez-Gimeno C, Raso J (2024) Pulsed electric fields effects on proteins: extraction, structural modification, and enhancing enzymatic activity. Bioelectricity 6(3):154. 10.1089/bioe.2024.0023 39372091 PMC11447477

[CR40] Meneses N, Jaeger H, Knorr D (2011) Minimization of thermal impact by application of electrode cooling in a co-linear PEF treatment chamber. J Food Sci 76(8):E536–E543. 10.1111/j.1750-3841.2011.02368.x 22417588

[CR41] Nadeeshani H, Hassouna A, Lu J (2022) Proteins extracted from seaweed *Undaria pinnatifida* and their potential uses as foods and nutraceuticals. Crit Rev Food Sci Nutr 62(22):6187–6203. 10.1080/10408398.2021.1898334 33703974

[CR42] O’ Connor J, Meaney S, Williams GA, Hayes M (2020) Extraction of protein from four different seaweeds using three different physical pre-treatment strategies. Molecules 25(8):2005. 10.3390/molecules25082005 32344706 PMC7221823

[CR43] Park JS, Han JM, Park SW, Kim JW, Choi MS, Lee SM, Haq M, Zhang W, Chun BS (2024) Subcritical water extraction of *Undaria pinnatifida*: comparative study of the chemical properties and biological activities across different parts. Mar Drugs 22(8):344. 10.3390/md22080344 39195460 PMC11355683

[CR44] Pataro G, Goettel M, Straessner R, Gusbeth C, Ferrari G, Frey W (2017) Effect of PEF treatment on extraction of valuable compounds from microalgae *C. vulgaris*. Chem Eng Trans 57:67–72. 10.3303/CET1757012

[CR45] Pedro B, Guedes L, André R, Gaspar H, Vaz P, Ascensão L, Melo R, Luísa Serralheiro M (2021) *Undaria pinnatifida* (*U. Pinnatifida*) bioactivity: antioxidant, gastro-intestinal motility, cholesterol biosynthesis and liver cell lines proteome. J Funct Foods 83:104567. 10.1016/j.jff.2021.104567

[CR46] Perez B, Baumgartner J, Macken D, Haberkorn I, Mathys A (2026) Pulsed electric fields for emerging single-cell bioprocessing in food applications: electropermeabilization mechanisms and design principles. Compr Rev Food Sci Food Saf 25(2):e70411. 10.1111/1541-4337.70411 41696771 PMC12908215

[CR47] Picot-Allain C, Mahomoodally MF, Ak G, Zengin G (2021) Conventional versus green extraction techniques—a comparative perspective. Curr Opin Food Sci 40:144–156. 10.1016/j.cofs.2021.02.009

[CR48] Pliquett U (2003) Joule heating during solid tissue electroporation. Med Biol Eng Comput 41(2):215–219. 10.1007/BF02344892 12691444

[CR49] Polikovsky M, Fernand F, Sack M, Frey W, Müller G, Golberg A (2016) Towards marine biorefineries: selective proteins extractions from marine macroalgae *Ulva* with pulsed electric fields. Innov Food Sci Emerg Technol 37:194–200. 10.1016/j.ifset.2016.03.013

[CR50] Postma PR, Cerezo-Chinarro O, Akkerman RJ, Olivieri G, Wijffels RH, Brandenburg WA, Eppink MHM (2018) Biorefinery of the macroalgae *Ulva lactuca*: extraction of proteins and carbohydrates by mild disintegration. J Appl Phycol 30:1281–1293. 10.1007/s10811-017-1319-8 29755208 PMC5928186

[CR51] Ranjha MMAN, Kanwal R, Shafique B, Arshad RN, Irfan S, Kieliszek M, Kowalczewski PŁ, Irfan M, Khalid MZ, Roobab U, Aadil RM (2021) A critical review on pulsed electric field: a novel technology for the extraction of phytoconstituents. Molecules 26(16):4893. 10.3390/molecules26164893 34443475 PMC8400384

[CR52] Raso J, Frey W, Ferrari G, Pataro G, Knorr D, Teissie J, Miklavčič D (2016) Recommendations guidelines on the key information to be reported in studies of application of PEF technology in food and biotechnological processes. Innov Food Sci Emerg Technol 37:312–321. 10.1016/j.ifset.2016.08.003

[CR53] Solana V, Ixtaina V, Dellatorre FG (2025) Postharvest processing of *Undaria pinnatifida*: effect of blanching and salting parameters on the quality of yudoshi-enzo wakame. Algal Res 90:104178. 10.1016/j.algal.2025.104178

[CR54] Steinbruch E, Wise J, Levkov K, Chemodanov A, Israel Á, Livney YD, Golberg A (2023) Enzymatic cell wall degradation combined with pulsed electric fields increases yields of water-soluble-protein extraction from the green marine macroalga *Ulva* sp. Innov Food Sci Emerg Technol 84:103231. 10.1016/j.ifset.2022.10323

[CR55] Steinbruch E, Kashyap M, Chemodanov A, Levkov K, Golberg A (2024) Continuous pulsed electric field processing for intensification of aqueous extraction of protein from fresh green seaweed *Ulva* sp. biomass. Food Hydrocoll 157:110477. 10.1016/j.foodhyd.2024.110477

[CR56] Taha A, Casanova F, Šimonis P, Stankevič V, Gomaa MAE, Stirkė A (2022) Pulsed electric field: fundamentals and effects on the structural and techno-functional properties of dairy and plant proteins. Foods 11(11):1556. 10.3390/foods11111556 35681305 PMC9180040

[CR57] Timmermans R, Mastwijk H, Berendsen L, Nederhoff A, Matser A, Van Boekel M, Nierop Groot M (2019) Moderate intensity Pulsed Electric Fields (PEF) as alternative mild preservation technology for fruit juice. Int J Food Microbiol 298:63–73. 10.1016/j.ijfoodmicro.2019.02.015 30925357

[CR58] Toepfl S, Heinz V, Knorr D (2007) High intensity pulsed electric fields applied for food preservation. Chem Eng Process Process Intensif 46(6):537–546. 10.1016/j.cep.2006.07.011

[CR59] Tomasevic I, Heinz V, Djekic I, Terjung N (2023) Pulsed electric fields and meat processing: latest updates. Ital J Anim Sci 22(1):857–866. 10.1080/1828051X.2023.2206834

[CR60] Vaessen EMJ, Timmermans RAH, Tempelaars MH, Schutyser MAI, den Besten HMW (2019) Reversibility of membrane permeabilization upon pulsed electric field treatment in *Lactobacillus plantarum* WCFS1. Sci Rep 9(1):19990. 10.1038/s41598-019-56299-w 31882651 PMC6934533

[CR62] Wang Q, Li Y, Sun DW, Zhu Z (2018) Enhancing food processing by pulsed and high voltage electric fields: principles and applications. Crit Rev Food Sci Nutr 58(13):2285–2298. 10.1080/10408398.2018.1434609 29393667

[CR61] Wang L, Hu Y, Zeng X, Wang R, Xu F (2026) Pulsed electric field technology in fruit and vegetable products processing: a non-thermal path to preserving quality and enhancing efficiency. J Future Foods. 10.1016/j.jfutfo.2026.05.007. in press

[CR63] Zhao W, Ma R, Sun Y, Xia Q, Wu Z, Fan X, Pan D (2026) Pulsed electric field processing of meat: mechanisms, quality responses and future perspectives. J Future Foods. in press 10.1016/j.jfutfo.2026.05.017

[CR64] Zhou Q, Wang Z, Chen W, Liu X, Cheong K, Zou Y, Zhong S, Li R (2024) The structural characteristics, biological activities and mechanisms of bioactive brown seaweed polysaccharides: a review. J Funct Foods 119:106303. 10.1016/j.jff.2024.106303

